# Integrated single-cell sequencing for the development of a GJA4-based precision immuno-prognostic model in melanoma

**DOI:** 10.1016/j.tranon.2025.102450

**Published:** 2025-07-09

**Authors:** Yantao Ding, Si Xie, Wenyang Nie, Yun Bai, Tianyu Yao, Yixiao Wang, Jiajie Chen, Bo Liang, Yi Zhou, Hui Cheng, Zaixing Wang, Shengxiu Liu

**Affiliations:** a Department of Dermatology, The First Affiliated Hospital of Anhui Medical University, Hefei, Anhui 230022, China; b Key Laboratory of Dermatology (Anhui Medical University), Ministry of Education, Hefei, Anhui 230022, China; c Inflammation and Immune-Mediated Diseases Laboratory of Anhui Province, Hefei, Anhui 230022, China; d First Clinical Medical College, Shandong University of Traditional Chinese Medicine, Jinan 250000, China; e Affiliated Hospital of Shandong University of Traditional Chinese Medicine, Jinan 250000, China; f Department of plastic surgery, The First Affiliated Hospital of Anhui Medical University, Hefei, Anhui 230022, China

**Keywords:** Melanoma, GJA4, Diagnosis, Prognosis, Tumor-associated endothelial cells, Personalized therapy

## Abstract

**Methods:**

We conducted an analysis of RNA-seq and microarray data obtained from the TCGA and GEO databases, alongside single-cell RNA sequencing (scRNA-seq) data from glioma patients within the GEO repository. This comprehensive investigation, augmented by experimental studies, concentrated on exploring the interactions between tumor-associated endothelial cells (TECs) and tumors, as well as elucidating the molecular mechanisms involved.

**Results:**

Single-cell sequencing analysis identified differentially expressed genes within tumor-associated endothelial cells. Further investigation highlighted GJA4 as a pivotal marker gene for a terminal subpopulation, with its expression linked to poor prognosis. Subsequent experiments were conducted to explore its underlying functional mechanisms.

**Conclusions:**

GJA4 is highly expressed in melanoma patients, and its differential expression in tumor-associated endothelial cells influences melanoma proliferation and migration. GJA4-based risk models hold potential as predictive and therapeutic targets for personalized melanoma treatment.

## Highlights

• Endothelial cell play an essential role in tumor microenvironment of melanoma.
• 2.GJA4 Significantly differentially expressed in tumor-associated endothelial cell.
• GJA4 expression in tumor endothelial cells may inhibit the accumulation of CD8+ T cells.

## Introduction

Melanoma, a highly aggressive skin cancer derived from melanocytes, has seen a significant rise in incidence in recent years, particularly in Western countries [1,2]. Epidemiological data indicate an annual increase in melanoma incidence of approximately 5–7 %, with an even higher growth rate observed among younger populations [3,4]. Pathologically, melanoma is marked by its aggressive invasiveness and a high propensity for metastasis, frequently spreading via the lymphatic system and bloodstream to distant organs such as the lungs [5], liver [6], and brain [7]. While early-stage melanoma can be effectively treated with surgical excision, metastatic melanoma presents limited therapeutic options [8]. Current treatment modalities, including immunotherapy and targeted therapy, are frequently associated with significant side effects and are only efficacious in a subset of patients [9,10]. Consequently, there is a pressing need to devise new therapeutic approaches to enhance survival outcomes and quality of life for patients with advanced melanoma.

The GJA4 protein, also known as Connexin 37 (Cx37), is a critical component of gap junction channels, facilitating intercellular communication and signal transduction [11]. In various malignancies, the expression and function of GJA4 have been closely linked to tumorigenesis and progression [12]. SResearch has shown that GJA4 may play a dual role in certain cancers, with the potential to both inhibit tumor growth and promote tumor cell migration and invasion. Specifically regarding melanoma, the role of GJA4 remains incompletely understood, but preliminary findings suggest that its aberrant expression in melanoma cells may be associated with increased tumor invasiveness and metastatic potential [13]. In recent years, research on GJA4 as a potential therapeutic target has gained traction, focusing on modulating its expression and function to impede tumor progression [11]. Although these studies are still in their early stages, targeted therapeutic strategies aimed at GJA4 hold promise for providing new treatment options for melanoma patients.

Single-cell sequencing technology, as a revolutionary advancement, has significantly propelled the development of personalized medicine [14,15]. By enabling comprehensive analysis of individual cells at the genomic, transcriptomic, and epigenomic levels, this technology unveils cellular heterogeneity with unprecedented resolution, facilitating the identification of key molecules and cell types [16,17]. In personalized medicine, single-cell sequencing is widely employed for precise disease diagnosis and the design of individualized treatment plans [18,19]. For instance, by analyzing single cancer cells within a tumor, researchers can discern tumor heterogeneity and evolutionary pathways, thereby formulating more accurate therapeutic strategies. Particularly in oncology, single-cell sequencing not only aids in the discovery of novel biomarkers and therapeutic targets but also monitors cellular responses and resistance mechanisms during treatment, thereby optimizing therapeutic outcomes and enhancing patient survival and quality of life. Consequently, single-cell sequencing technology holds vast potential in personalized medicine, especially in cancer therapy, and is rapidly becoming an indispensable tool in precision medicine.

## Methods

### Data sources and acquisition

Single-cell RNA sequencing (scRNA-seq) data was accessed from the Gene Expression Omnibus (GEO) database under accession number GSE189889 (https://www.ncbi.nlm.nih.gov/geo/). Bulk RNA-seq data was retrieved from The Cancer Genome Atlas (TCGA) through the official GDC portal (https://portal.gdc.cancer.gov/).

### Data processing and cell type identification

Each dataset was processed using the Seurat package (v4.3.0) in R (v4.2.2). Initially, potential doublets were excluded using the DoubletFinder package (v2.0.3), and low-quality cells were filtered out, maintaining cell quality within the following ranges: 300 < nFeature < 6000 and 500 < nCount < 100,000. Cells meeting these criteria were retained for further analysis. Additionally, cells were required to have <25 % mitochondrial gene expression and <5 % red blood cell gene expression to be included in subsequent analyses.

Next, the expression matrix was normalized, and the top 2000 highly variable genes (HVGs) were identified and standardized. PCA analysis was then performed on these genes. To address batch effects across samples, the Harmony package (v0.1.1) was utilized, selecting the top 30 principal components (PCs) for dimensionality reduction and clustering [20]. Following this, UMAP was employed to project the results onto a two-dimensional plot, facilitating cell type identification. Relevant cell markers from the literature were used to annotate cell clusters, thereby identifying distinct cell types and examining their distribution and proportions [21].

### Enrichment analysis of differentially expressed genes

Differentially expressed genes (DEGs) for each cell type were identified using the "FindAllMarkers" function with default settings, employing the Wilcoxon rank-sum test. Genes expressed in over 25 % of cells within clusters and exhibiting a log fold change (logFC) greater than 0.25 were selected. Enrichment analysis of differentially expressed genes (DEGs) was performed using the clusterProfiler package, with an emphasis on pathways related to each cell type as defined by Gene Ontology (GO) Biological Processes (BP).

### Assessment of cellular copy number variation (CNV) using infercnv

The InferCNV algorithm package (v1.17.0) was utilized to calculate CNV levels. Copy number karyotyping analysis was performed on non-diploid tumors to differentiate between non-tumor cells and malignant tumor cells. Endothelial cells were used as a reference in InferCNV to determine significant chromosomal copy number variations in cancer cells.

### Enrichment and AUCell analysis of differentially expressed genes (DEGs) in cellular subpopulations

Differentially expressed genes (DEGs) within various endothelial cell subpopulations were identified using the "FindAllMarkers" function in conjunction with the Wilcoxon rank-sum test. GO-BP enrichment analysis was subsequently carried out on these genes using the clusterProfiler package. AUCell analysis was employed to detect cells exhibiting active gene sets within the scRNA-seq data, followed by functional annotation and visualization of Gene Ontology (GO) terms [22].

### Cell lineage and trajectory analysis

To assess the temporal differentiation and developmental dynamics of melanoma endothelial cell subpopulations, three software packages were used. The cytoTRACE algorithm package (v0.3.3) evaluated stemness in each subpopulation. The Monocle package (v2.24.0) constructed cell differentiation trajectories using DDRTree for dimensionality reduction, allowing observation of developmental progression. Finally, Slingshot was employed to further analyze cell trajectories, using the getLineages function to fit a minimum spanning tree (MST) and the getCurves function to estimate lineage-specific gene expression over time.

### Cell-to-Cell communication

Within the context of single-cell RNA sequencing data, the CellChat package (v1.6.1) was utilized to predict interactions between cell types. The CellChatDB.human database was referenced for ligand-receptor interactions, with a significance cutoff of *P* < 0.05 to predict intercellular communication.

### Construction of a prognostic model for melanoma endothelial cells

To assess the prognostic significance of endothelial cell-associated genes in melanoma, key marker genes specific to this endothelial subpopulation were identified. Univariate Cox analysis and Lasso regression were conducted using the "survival" R package to pinpoint significant prognostic genes. These were subsequently incorporated into a multivariable Cox model to develop a prognostic signature. The risk score for each sample was then calculated using the derived formula: Risk Score=∑(Expression level of Genei × Coefficienti)\text{Risk Score} = \sum (\text{Expression level of Gene}_i \times \text{Coefficient}_i)Risk Score=∑(Expression level of Genei​ × Coefficienti​) Samples were stratified into high-risk and low-risk categories according to the median risk score. Kaplan-Meier survival analysis was performed to evaluate prognostic outcomes, utilizing the timeROC package (v0.4.0) was used to generate ROC curves for 1, 3, and 5-year intervals. The correlation between model genes, risk scores, and overall survival (OS) was further examined.

### Predictions of column line chart

Considering the importance of risk gene scores and risk cell type scores as independent prognostic factors, multivariate Cox regression analysis was conducted, incorporating both clinical and pathological variables. A column line chart was created to forecast the 1-year, 3-year, and 5-year overall survival rates for melanoma patients within the TCGA cohort. Separate Cox regression models were used for T, N, and M staging, risk scores, and age groups, with corresponding column line charts generated. The charts were generated using the rms package (v6.7–0), and calibration plots were employed to evaluate predictive accuracy. The model's performance was additionally assessed using the C-index score.

### Assessment of tumor-infiltrating immune cells

Immune-related scores were derived using the CIBERSORT (v0.1.0), ESTIMATE (v1.0.13), and xCell (v1.1.0) packages to provide a comprehensive assessment of the immune microenvironment in cancer patients. Furthermore, the levels of 22 immune cell types identified by the CIBERSORT algorithm were evaluated across different groups. Their correlations with risk scores, model genes, and overall survival (OS) were analyzed. Comparisons were conducted between the Stromal Score, Immune Score, ESTIMATE Score, and Tumor Purity across different groups.

### Data variations and enrichment analysis in prognostic models

Differential analysis of high- and low-risk groups was conducted using the DESeq2 package (v1.38.1), with significance defined as *P* < 0.05P < 0.05P < 0.05. Subsequently, GO, KEGG, and GSEA were performed on the differentially expressed genes using the clusterProfiler package.

### Analysis of somatic mutation

Somatic mutation data were retrieved from the TCGA database, and the frequency of mutations in both highly mutated genes and model genes was analyzed utilizing the maftools package (v2.14.0). Tumor mutation burden (TMB) was assessed for each glioblastoma sample. Based on the median TMB value, samples were categorized into high TMB and low TMB groups. Kaplan-Meier survival analysis was performed on the four groups created by integrating TMB status with high-risk and low-risk scores. Additionally, copy number variations (CNVs) of the model genes were analyzed to investigate their genomic alterations.

### Predicting the immunotherapy response of chemotherapy in cancer treatment

The pRRophetic package was utilized to estimate the half-maximal inhibitory concentration (IC50) for chemotherapy agents pertinent to melanoma treatment [23]. Sensitivity to these drugs across different groups was assessed to predict their immunotherapeutic effects [24].

### Cell collection and culture

The cell lines utilized in this study—HaCaT, A375, WM-115, and HUVECs—were sourced from ScienCell Research Laboratories and maintained in DMEM medium (Gibco BRL, USA). The cells were cultured in a humidified incubator at 37 °C with 5 % CO2, using Endothelial Cell Medium (ScienCell Research Laboratories). The medium was supplemented with 1 % endothelial cell growth factor, 5 % fetal bovine serum, and 1 % antibiotic solution. The medium was refreshed every three days, and cells between passages 3 and 6 were used for experimental procedures. Upon reaching confluence, the cells were dissociated using either enzymatic or non-enzymatic methods and transferred to new culture flasks at an appropriate density to promote adequate proliferation for subsequent experiments.

### Cell transfection and lentivirus vector construction and cell infection

Two siRNAs targeting GJA4 were synthesized by Ribobio (Guangzhou, China). Transfections were performed using Lipofectamine 3000 (Invitrogen, USA) according to the manufacturer’s guidelines. The sequences of the GJA4-targeting siRNAs are provided in ***Supplementary Table 1***. Recombinant lentiviral and control vectors were generated by GenePharma (Shanghai, China). Lentiviral infection was carried out by adding the virus solution to the cells with 5 μg/mL polybrene (Sigma-Aldrich). Following a 72-hour infection period, cells were selected with 2 μg/mL puromycin. Puromycin-resistant cells were then collected and cultured to establish stable cell lines.

### Quantitative RT-PCR analysis

Total RNA was isolated from cells or tissues using the RNeasy Mini Kit (Qiagen). RNA concentration and purity were assessed using a NanoDrop spectrophotometer. cDNA synthesis was performed with the High-Capacity cDNA Reverse Transcription Kit (Applied Biosystems) according to the manufacturer's instructions. Quantitative RT-PCR was conducted using SYBR Green PCR Master Mix (Applied Biosystems) and specific primers for target genes. PCR reactions were run in triplicate on a real-time PCR system (Applied Biosystems 7500) with an initial denaturation at 95 °C for 10 min, followed by 40 cycles of 95 °C for 15 s and 60 °C for 1 min. Gene expression levels were normalized to GAPDH Primer sequences, synthesized by Tsingke Biotech (Beijing, China), are provided in ***Supplementary Table 1***, and relative expression was calculated using the ΔΔCt method. Statistical significance was determined using appropriate statistical tests, with p-values < 0.05 considered significant.

### Immunohistochemistry

Formalin-fixed, paraffin-embedded tissue specimens were sectioned at 4 μm and placed on glass slides. The sections were deparaffinized with xylene and subsequently rehydrated through a graded ethanol series. Antigen retrieval was achieved by incubating the sections in citrate buffer (pH 6.0) using a microwave for 15 min. Endogenous peroxidase activity was inhibited by treating the sections with 3 % hydrogen peroxide in methanol for 10 min. Non-specific binding was then blocked using 5 % bovine serum albumin for 30 min. The sections were incubated overnight at 4 °C with primary antibodies targeting GJA4 (1:200, Abcam) and Ki67 (1:100, Abcam). Following incubation, biotinylated secondary antibodies were applied for 30 min. The VECTASTAIN Elite ABC Kit was used for detection, and color development was achieved with DAB substrate. Counterstaining was performed with hematoxylin, followed by dehydration, clearing in xylene, and mounting. The staining was assessed under a light microscope, and the proportion of positively stained cells was determined from five high-power fields. Statistical analysis was performed with SPSS version 25.0, considering p-values < 0.05 as indicative of statistical significance.

### Angiogenesis assay

For the tube formation assay, Matrigel was applied to 96-well plates and allowed to polymerize at 37 °C. HUVECs were then seeded at a concentration of 2 × 10^4 cells/mL and exposed to test compounds or controls for 6–8 h. Tube formation was evaluated with an inverted microscope, and the resulting images were analyzed using ImageJ software to measure total tube length and count branch points. Statistical significance was determined using one-way ANOVA with subsequent Tukey's post hoc test, considering p-values < 0.05 as indicative of significance.

### Wound healing

Wound healing assays were performed using a standardized scratch wound model. Confluent monolayers of cells were scratched using a sterile pipette tip to create a uniform wound gap. The cells were then washed to remove debris and incubated in fresh medium. Wound closure was monitored at specified time points (e.g., 0, 24, 48 h) using a phase-contrast microscope. Images of the wound area were captured, and the extent of healing was quantified by measuring the remaining wound area using ImageJ software. Data were expressed as the percentage of wound closure relative to the initial wound area. Statistical analysis was performed using appropriate tests, with p-values < 0.05 considered statistically significant.

### The Transwell experiment

Cell migration was evaluated using Transwell chambers. A total of 2 × 10⁴ cells were plated in 200 μL of serum-free medium in the upper compartment of each well. Some wells were treated with Matrigel solution to assess migration under different conditions, while others were left untreated. After a 48-hour incubation, the chambers were retrieved, and cells were fixed with 4 % paraformaldehyde (PFA) and stained with 0.1 % crystal violet (Solarbio, China). The number of migratory cells was counted under a light microscope, and images were captured and analyzed.

### The experiment of mIHC

Formalin-fixed, paraffin-embedded tissue sections (4 μm) were subjected to deparaffinization, rehydration, and antigen retrieval in citrate buffer (pH 6.0). Endogenous peroxidase activity was inhibited using 3 % hydrogen peroxide in methanol, while non-specific binding was minimized with 5 % bovine serum albumin in PBS. The sections were incubated overnight at 4 °C with primary antibodies against GJA4 (1:200), CD31 (1:100), and CD8 (1:100). Following this, fluorophore-conjugated secondary antibodies (Alexa Fluor 488, 594, and 647) were applied for 1 h at room temperature. The sections were counterstained with DAPI and mounted in a fluorescence-compatible medium. Confocal microscopy was used for imaging, and the data were analyzed with ImageJ and Zen software. Statistical significance was determined using one-way ANOVA with Tukey’s post hoc test, with p-values < 0.05 considered significant.

### The animal experiment

In the metastasis model, 18 mice were randomly divided into three groups: sh-GJA4-NC, sh-GJA4–1, and sh-GJA4–2 (*n* = 6/group). Stable LN229 and U251 cells (1 × 10^6 cells/100 μL of PBS) were intravenously injected into the tail vein of each mouse. After 5 weeks, distant metastasis was assessed using an IVIS imaging system. The study received approval from the Anhui Animal Care and Use Committee at Anhui Medical University.

### Statistical analysis

Biomedical analyses were conducted using R software version 4.1.3, and experimental data were analyzed with GraphPad Prism version 8.0. Mean values and standard deviations were derived from three independent experiments. Comparisons between two groups were made using Student's *t*-test, while one-way ANOVA with Tukey’s post hoc test was used for multiple group comparisons. Statistical significance was denoted as follows: *p < 0.05, **p < 0.01, and ***p < 0.001.

## Results

### Cell type identification in melanoma

Following initial quality control using R software, 35,386 high-quality cells were retained for analysis. A UMAP plot (Supplementary Figure 1A, left) visualized the melanoma cells from nine samples post batch effect removal, while dimensionality reduction clustering identified 33 distinct cell clusters (Supplementary Figure 1A, right). Subsequently, another UMAP plot (Supplementary Figure 1B) illustrated the tissue origin of all cells and their distribution across the cell cycle.

Marker gene expression was used to classify the 35,386 cells into nine cell types: melanoma cells (18,083), NK_T cells (10,548), endothelial cells (1418), B_Plasma cells (1295), myeloid cells (1535), fibroblasts (1651), pericytes (552), epithelial cells (225), and mast cells (79). The UMAP plots (Supplementary Figure 1C, D) further depicted the distribution of these cell types across different samples, tissue origins, and cell cycle phases.

Among the samples, four were classified as "Mets" and five as "Primary," with bar graphs and box plots (Supplementary Figure 1E, F) illustrating the proportions and distributions of the nine cell types across patients. A bubble plot (Supplementary Figure 1 G) highlighted the differential expression of the top 10 marker genes for each cell type.

A volcano plot (Supplementary Figure 1H) showed the expression levels of differentially expressed genes among the nine cell types, while word clouds (Supplementary Figure 1I) displayed the enrichment frequency of various genes for each cell type. Additionally, GO_BP enrichment analysis (Supplementary Figure 1 J) of the differentially expressed genes across the nine cell types highlighted their specific biological functions. For instance, endothelial cells were notably associated with processes such as ameboidal cell migration, epithelial cell migration, and epithelium migration, suggesting a more active role in tumor epithelial cell migration and related biological processes compared to other cell types.

### Identification of endothelial subtypes in melanoma

**Fig. 1:**
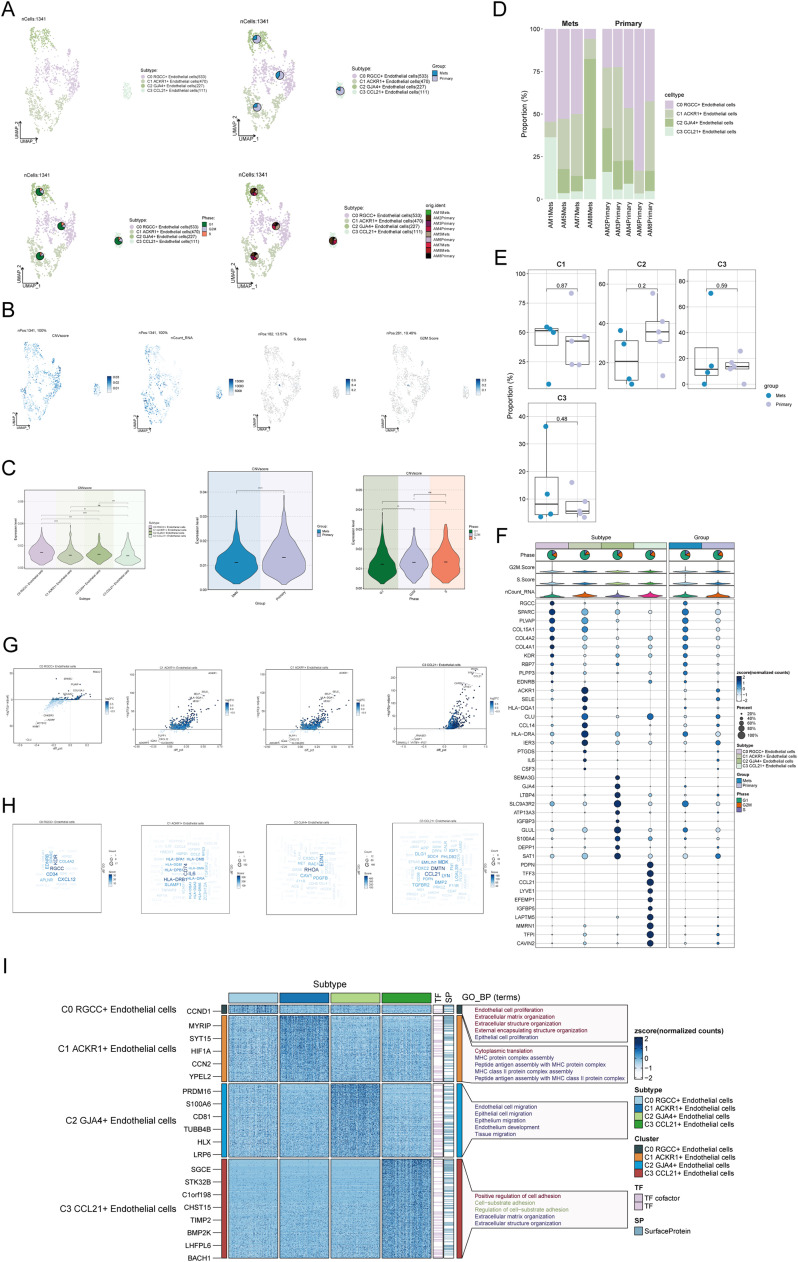
Identification of Endothelial Cell Subpopulations in Melanoma A: UMAP visualization showcased the distribution of 1341 endothelial cells divided into four cell subpopulations (top left), the tissue type of endothelial cell subpopulations (top right), cell cycle stages (bottom left), and cell tissue origins (bottom right). B: UMAP visualization exhibited the CNVscore, nCount_RNA, S.score, and G2M.score of 1341 endothelial cells. C: Violin plot displayed the CNVscore of endothelial cell subpopulations categorized by subpopulation type, tissue type origins, and cell cycle stages. D: Bar chart displayed the proportion of the four endothelial cell subpopulations across different sample origins. E: Box plot highlighted the distribution of the four endothelial cell subpopulations among different cell tissue origins. F: Bubble plot showed the differential expression of the top 10 marker genes for endothelial cell subpopulations. The color of the bubbles represented normalized counts, while the size represented the proportion of gene expression. G: Volcano plot presented the expression pattern of differentially expressed genes among the four endothelial cell subpopulations. H: Word cloud displayed the frequency of gene enrichment for different genes among the four endothelial cell subpopulations. I: Heatmap showed the top 5 enriched Gene Ontology Biological Process (GO-BP) terms for differentially expressed genes among the four endothelial cell subpopulations.

In our analysis of melanoma-associated endothelial cells, 1341 high-quality endothelial cells were retained after quality control and batch effect removal. UMAP clustering (Fig. 1A, top left) led to the identification of four distinct endothelial subtypes based on marker gene annotation. UMAP plots and pie charts (Fig. 1A) illustrated the proportions of tissue types, cell cycle phases, and tissue origins within each endothelial subtype.

To examine the distribution across tissue origins, a bar graph (Fig. 1D) showed the proportions of the four endothelial subtypes in different tissue types, while a box plot (Fig. 1E) visualized their distribution across different tissue types. Subtypes C0 RGCC+ and C1 ACKR1+ Endothelial cells were more prevalent in both "Mets" and "Primary" samples, whereas subtypes C2 GJA4+ and C3 CCCL21+ Endothelial cells were less common.

A bubble plot (Fig. 1F) illustrated the differential expression of the top 10 marker genes across each endothelial subtype, while a volcano plot (Fig. 1G) showcased the distinct expression patterns of these marker genes. Word cloud visualizations (Fig. 1H) emphasized the enrichment frequency of different genes within each of the four endothelial subtypes.

To further distinguish the endothelial subtypes, we analyzed the overall distribution of CNV scores, nCount RNA, S.Score, and G2M.Score within each subtype (Fig. 1B). Notably, C0 RGCC+ Endothelial cells exhibited relatively higher CNV scores, while subtypes C1 ACKR1+ and C3 CCCL21+ Endothelial cells showed elevated nCount RNA values. By analyzing copy number variation (CNV) patterns, insights were gained into tumor cell heterogeneity and evolution. CNV scores for each endothelial subtype were compared (Fig. 1C), revealing that C0 RGCC+ and C2 GJA4+ Endothelial cells had higher CNV scores.

Additionally, CNV scores were assessed across different tissue types and cell cycle phases of melanoma endothelial cells, with primary samples and S and G2M phases showing higher CNV scores. Finally, differentially expressed genes among the four endothelial subtypes were selected for enrichment analysis to elucidate their associated biological processes, molecular functions, and cellular components (Fig. 1I). For instance, C0 RGCC+ Endothelial cells were enriched in processes like endothelial cell proliferation, extracellular matrix organization, and epithelial cell proliferation. C1 ACKR1+ Endothelial cells were associated with cytoplasmic translation, the MHC protein complex assembly and peptide antigen assembly with the MHC protein complex were noted. C2 GJA4+ endothelial cells were associated with processes such as endothelial cell migration, epithelial cell migration, and tissue migration. In contrast, C3 CCL21+ endothelial cells were predominantly involved in the positive regulation of cell adhesion, cell-substrate adhesion, and extracellular matrix organization.

### Pseudotemporal analysis using cytotrace and monocle

To explore the heterogeneity among endothelial cell subtypes in melanoma, we utilized CytoTrace and Monocle for pseudotemporal analysis. CytoTrace identified C0 RGCC+ Endothelial cells as the most differentiated subtype, while C1 ACKR1+ Endothelial cells displayed the least differentiation (Supplementary Figure 2A, B). These findings were supported by UMAP, violin, and ridge plots (Supplementary Figure 2C-E).

Using the Monocle algorithm, we generated differentiation trajectories for the endothelial subtypes, which highlighted the progression of differentiation. C0 RGCC+ Endothelial cells predominantly clustered in the late differentiation stages, showing the highest degree of differentiation, while C1 ACKR1+ Endothelial cells were mostly in the earliest stage (Supplementary Figure 2E). This finding was corroborated by the CytoTrace analysis, which revealed that the trajectory of marker gene expression for these subtypes aligned with this pattern (Supplementary Figure 2F).

Bar plots further revealed that state 3 consisted exclusively of C1 ACKR1+ Endothelial cells, which were also dispersed across all nine states. In contrast, C0 RGCC+ Endothelial cells were primarily present in states 4, 5, and 6, while C3 CCCL21+ Endothelial cells had a smaller distribution across all states. Comparatively, the proportions of C0 and C1 subtypes were the largest, with C3 being the smallest (Supplementary Figure 2 G).

A heatmap illustrated the expression levels of the top 10 marker genes along the trajectory (Supplementary Figure 2H). Marker genes for C0 RGCC+ Endothelial cells showed high expression predominantly in the late stages. In contrast, marker genes for C1 ACKR1+ and C3 CCCL21+ Endothelial cells peaked during the mid-stages, and marker genes for C2 GJA4+ Endothelial cells were highly expressed both at the initial and late stages of the trajectory.

### Slingshot analysis of endothelial cell subtypes

To further dissect the developmental trajectories of endothelial cell subtypes, we applied the Slingshot method to fit differentiation trajectories. Two distinct lineage trajectories were identified: Lineage 1, which progressed from C3 CCCL21+ Endothelial cells to C0 RGCC+ Endothelial cells and finally to C1 ACKR1+ Endothelial cells; and Lineage 2, which also initiated with C3 CCCL21+ Endothelial cells, progressed through C0 RGCC+ Endothelial cells, and terminated in C2 GJA4+ Endothelial cells. UMAP plots depicted these differentiation trajectories (Supplementary Figure 3A-C), with C3 CCCL21+ Endothelial cells at the starting points and C1 ACKR1+ and C2 GJA4+ Endothelial cells at the respective terminal stages, indicating their higher degrees of differentiation.

GO_BP enrichment analysis of these subtypes (Supplementary Figure 3D) identified pathways enriched in processes such as adhesion, cell-substrate interaction, development, signaling pathways, cell cycle, and apoptosis. These included specific pathways like insulin-like growth factor signaling, platelet aggregation, endothelial contraction, and smooth muscle cell growth.

Scatter plots were utilized to monitor the expression variations of key marker genes across the two differentiation trajectories, showing that CCL21 was prominent early on, while ACKR1 and GJA4 were more prevalent at later stages. RGCC had a significant presence during the intermediate differentiation phase, consistent with earlier findings (Supplementary Figure 3E).

### Cell-Cell communication in endothelial cells

To investigate intercellular interactions and ligand-receptor communication networks, we employed the "CellChat" package to analyze cell communication within scRNA-seq data. We initially established intercellular communication networks among different cell types, quantifying both the number of interactions (edges) and the strength of these interactions (edge thickness) (Supplementary Figure 4A).

To identify key signals associated with the four endothelial subtypes, we used CellChat to quantify the ligand-receptor networks and predict critical incoming and outgoing signals. In melanoma, cell types can act as either signal senders (releasing cytokines or ligands) or receivers (targeting these signals through receptors). This ligand-receptor communication plays a significant role in melanoma development. Our analysis showed that myeloid cells had the highest incoming interaction strength, while fibroblasts exhibited the highest outgoing interaction strength. Notably, C3 CCCL21+, C1 ACKR1+, and C2 GJA4+ Endothelial cells displayed high levels of both incoming and outgoing interaction strength (Supplementary Figure 4B).

Beyond individual pathways, we also examined global communication patterns among cell populations and their coordinated functions. Using a pattern recognition approach based on non-negative matrix factorization, we identified communication patterns linking cell populations with signaling pathways, both in terms of sending (output signals) and receiving (input signals). C3 CCCL21+ Endothelial cells showed stronger incoming communication, while C0 RGCC+, C1 ACKR1+, and C2 GJA4+ Endothelial cells exhibited stronger outgoing communication (Supplementary Figure 4C-D).

Particularly, C2 GJA4+ Endothelial cells were found to have robust outgoing and incoming signal strengths. We further quantified the number and strength of interactions between C2 GJA4+ Endothelial cells and other cell types (Supplementary Figure 4E), revealing extensive communication with other endothelial subtypes and tumor cells. These interactions suggest that fibroblast subtypes and tumor cells may engage in prominent receptor-ligand interactions. We further analyzed communication between fibroblast subtypes and tumor cells, revealing close associations between the C3 subpopulation and melanoma fibroblasts, as well as between fibroblasts and the different endothelial subtypes (Supplementary Figure 4F).

### Identification of prognostic marker genes for C2 GJA4+ endothelial cells

**Fig. 2:**
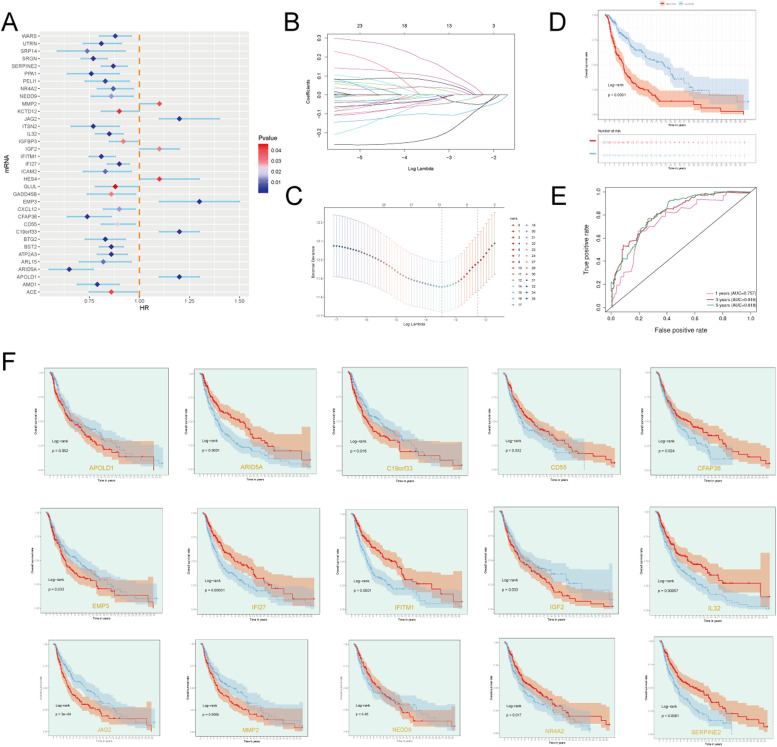
**Selection of Prognostic-Related Key Genes** A: The forest plot displayed the results of univariate Cox analysis, with *P* ≤ 0.05. The null line represents HR=1, HR<1 indicates a protective factor, and HR>1 indicates a risk factor. B-C: A total of 15 genes, selected through lasso regression, comprised the risk score. The lambda plot demonstrated this result (lambda.min=0.032). D: Kaplan-Meier survival curves illustrated the survival differences between High C2 Score and Low C2 Score groups. E: The ROC curve graph presented the AUC scores for 1 year, 3 years, and 5 years (1 year=0.757, 3 years=0.816, 5 years=0.818). F: Survival curves based on each modeling gene were shown (red denotes high expression group, blue denotes low expression group). Fig. 7: Identification and Analysis of Related Genes for C2 GJA4+ Endothelial Cells A: The curve plot depicted the hazard scores for High C2 Score and Low C2 Score groups (top), the scatter plot displayed the events of survival/death over time for High C2 Score and Low C2 Score (middle), and the heatmap demonstrated the differential gene expression between High C2 Score and Low C2 Score, with color scale based on standardized data (bottom). Blue represented the low-risk score group, while red represented the high-risk score group. B: The correlation of four genes with C2 Score. C19orf33, EMP3, APOLD1, and JAG2 exhibited a positive correlation with Risk. C: Peak plots and box plots showcased the expression differences of the four genes between High C2 Score and Low C2 Score groups. D-E: Scatter plots displayed the correlation analysis between the four genes and overall survival (OS) and the correlation analysis among the four genes. F: Box plots demonstrated the differential expression of the four genes between High C2 Score and Low C2 Score groups, as well as across different age groups (elderly and young), various ethnicities (Asian, Black, or Caucasian), and different TNM stages (T0, T1, T2, T3, T4, Ti; N0, N1, N2, N3, M0, M1). *p ≤ 0.05, **p < 0.01, ***p < 0.001 indicated significant differences, and "ns" indicated no significant difference.

We began our analysis with univariate Cox regression to evaluate the prognostic significance of the top 100 marker genes for C2 GJA4+ endothelial cells, identifying 35 genes significantly associated with patient outcomes (*p* < 0.05) (Fig. 2A). To refine the gene set and address potential multicollinearity, we applied LASSO regression, which selected 15 genes with prognostic significance (Fig. 2B-C).

Subsequently, we performed multivariate Cox regression on these 15 genes to calculate their respective hazard coefficients. The risk score for each gene was derived using the formula: Risk Score=(C19orf33 × 0.136854)+(IGF2 × 0.071299)+(EMP3 × 0.264124)+(IFI27 × −0.06348)+(SERPINE2 × −0.0664)+(MMP2 × 0.011703)+(IFITM1 × −0.08451)+(JAG2 × 0.048147)+(ARID5A × −0.28372)+(CFAP36 × −0.02797)+(IL32 × −0.06765)+(NEDD9 × −0.02665)+(NR4A2 × −0.02995)+(CD55 × −0.07263)+(APOLD1 × 0.113197)\text{Risk Score} = (\text{C19orf33} \times 0.136854) + (\text{IGF2} \times 0.071299) + (\text{EMP3} \times 0.264124) + (\text{IFI27} \times −0.06348) + (\text{SERPINE2} \times −0.0664) + (\text{MMP2} \times 0.011703) + (\text{IFITM1} \times −0.08451) + (\text{JAG2} \times 0.048147) + (\text{ARID5A} \times −0.28372) + (\text{CFAP36} \times −0.02797) + (\text{IL32} \times −0.06765) + (\text{NEDD9} \times −0.02665) + (\text{NR4A2} \times −0.02995) + (\text{CD55} \times −0.07263) + (\text{APOLD1} \times 0.113197)Risk Score=(C19orf33 × 0.136854)+(IGF2 × 0.071299)+(EMP3 × 0.264124)+(IFI27 × −0.06348)+(SERPINE2 × −0.0664)+(MMP2 × 0.011703)+(IFITM1 × −0.08451)+(JAG2 × 0.048147)+(ARID5A × −0.28372)+(CFAP36 × −0.02797)+(IL32 × −0.06765)+(NEDD9 × −0.02665)+(NR4A2 × −0.02995)+(CD55 × −0.07263)+(APOLD1 × 0.113197). Although the key subset of GJA4 was not directly included in the 15-gene risk score model, there may be a potential functional association between the two. On the one hand, functional enrichment and co-expression analysis showed that GJA4 and some of the model genes were involved in multiple pathways closely related to the occurrence and development of diseases, such as intercellular communication, inflammation and angiogenesis, suggesting that key subsets may indirectly affect the risk assessment ability of the model through these gene networks. On the other hand, regulatory network speculation suggests that GJA4 may be involved in regulating the expression activity of some modeled genes as an upstream regulator or a downstream effector molecule. In addition, single-cell expression profiling analysis further showed that some of the modeling genes had specific expression trends in key subsets with high GJA4 expression, strengthening the biological basis of this subset in the predictive ability of the model. Therefore, although GJA4 was not directly included in the modeling gene set, there may be indirect regulatory and functional synergies between the key subpopulation characteristics represented by GJA4 and the risk score model, which is worthy of further research and experimental verification. Patients were categorized into high-risk and low-risk groups based on the median risk score. Using the risk score model, patients were classified into high and low C2 GJA4+ endothelial cell score groups. Kaplan-Meier survival analysis indicated that patients in the low-score group had significantly improved overall survival (OS) compared to those in the high-score group (*p* < 0.0001) (Fig. 2D). To evaluate the predictive performance of the model, we computed the area under the curve (AUC) values from ROC curves for 1-year, 3-year, and 5-year overall survival (OS) in the TCGA cohort. The resulting AUC values were 0.757, 0.816, and 0.818, respectively (Fig. 2E). Among the 15 genes analyzed, C19orf33, EMP3, IGF22, and MMP2 were linked to notably improved survival rates in the low-score group compared to those in the high-score group (*p* < 0.05). Although APOLD1 showed a trend towards lower OS in the high-score group, it did not reach statistical significance and was excluded (Fig. 2F).

### Correlation analysis based on the established prognostic model

**Fig. 3:**
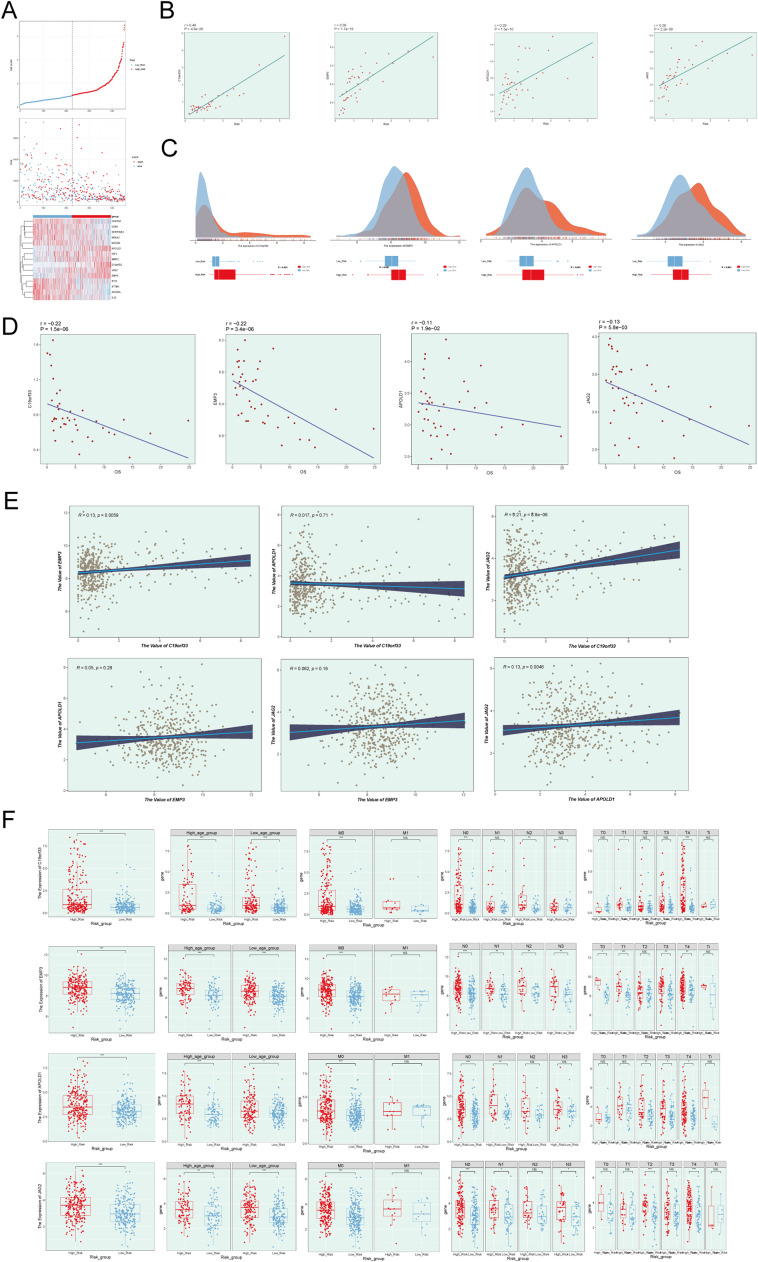
**Identification and Analysis of Related Genes for C2 GJA4+ Endothelial Cells** A: The curve plot depicted the hazard scores for High C2 Score and Low C2 Score groups (top), the scatter plot displayed the events of survival/death over time for High C2 Score and Low C2 Score (middle), and the heatmap demonstrated the differential gene expression between High C2 Score and Low C2 Score, with color scale based on standardized data (bottom). Blue represented the low-risk score group, while red represented the high-risk score group. B: The correlation of four genes with C2 Score. C19orf33, EMP3, APOLD1, and JAG2 exhibited a positive correlation with Risk. C: Peak plots and box plots showcased the expression differences of the four genes between High C2 Score and Low C2 Score groups. d-E: Scatter plots displayed the correlation analysis between the four genes and overall survival (OS) and the correlation analysis among the four genes. F: Box plots demonstrated the differential expression of the four genes between High C2 Score and Low C2 Score groups, as well as across different age groups (elderly and young), various ethnicities (Asian, Black, or Caucasian), and different TNM stages (T0, T1, T2, T3, T4, Ti; N0, N1, N2, N3, M0, M1). *p ≤ 0.05, **p < 0.01, ***p < 0.001 indicated significant differences, and "ns" indicated no significant difference.

Multivariable Cox regression analysis was performed to evaluate the prognostic significance of C2 GJA4+ endothelial cell gene signatures in conjunction with age, race, and tumor TNM stage. A nomogram was developed to combine these variables and forecast 1-year, 3-year, and 5-year overall survival (OS) in melanoma patients. Calibration curves were employed to assess and validate the accuracy of the model's predictions (Supplementary Figure5 A-C). The nomogram, incorporating age, risk group, and stage, indicated that the risk score had a substantial impact on predicting overall survival (OS) (Supplementary Figure5 E). Internal cross-validation within the TCGA cohort produced area under the curve (AUC) values for 1-year, 3-year, and 5-year OS predictions, as illustrated in Supplementary Figure5 D. Risk score distributions between the high and low C2 GJA4+ endothelial cell score groups were visualized through curve plots, while scatter plots depicted the distribution of survival and death events over time. Furthermore, a heatmap illustrated the differential expression of the 15 model genes between the two groups (Fig. 3A). Correlation analysis of the four model genes (C19orf33, EMP3, APOLD1, JAG2) revealed a positive association with risk scores and a negative association with overall survival (OS) (Figs. 3B and 7D). Pairwise correlation analysis revealed that all gene pairs were positively correlated, except for a negative correlation between APOLD1 and C19orf33 (Fig. 3E). Volcano and box plots further highlighted the differential expression of the four model genes between high and low C2 GJA4+ endothelial cell score groups, showing elevated expression levels in the low-score group (Fig. 3C). Box plots also demonstrated the differential expression of these genes with respect to age and tumor stage (TNM) between the two groups (Fig. 3F).

### Analysis of immune infiltration in high versus low C2 GJA4+ endothelial cell score groups

**Fig. 4:**
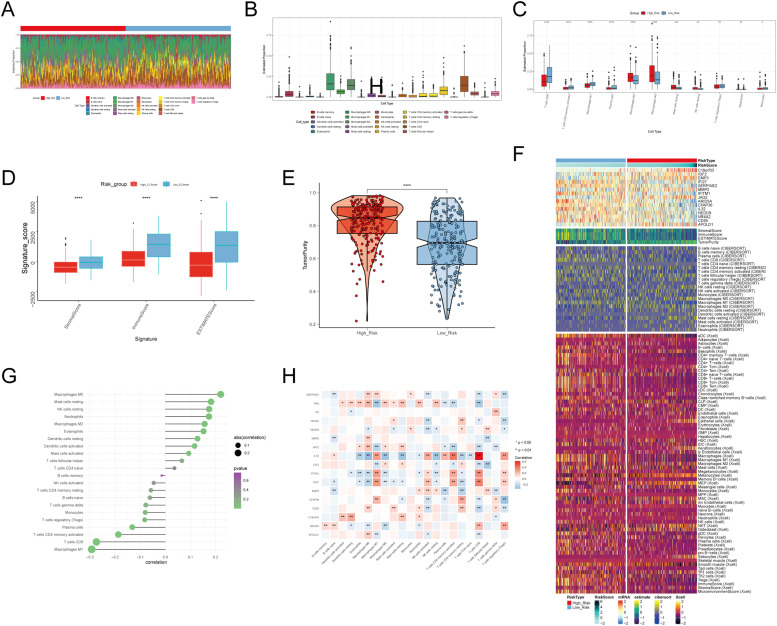
**Analysis of Immune Infiltration Differences between High C2 Score and Low C2 Score** A: The heatmap displayed the immune cell infiltration profiles for High C2 Score and Low C2 Score groups. Colors represented different cell types. B-C: Box plots illustrated the proportions of immune cell infiltration and the proportions of immune infiltrating cells in High C2 Score and Low C2 Score. The box plot showed the median (line within the box), upper and lower quartiles (box), and data range. D: Box plots demonstrated the differences in StroalScore, ImmuneScore, and ESTIMATEScore between High C2 Score and Low C2 Score. The box plot presented the median (line within the box), upper and lower quartiles (box), and data range. *p ≤ 0.05, **p < 0.01, ***p < 0.001 indicated significant differences, and "ns" indicated no significant difference. E: Box plots and violin plots showcased the differences in tumor purity between High C2 Score and Low C2 Score. *p ≤ 0.05, **p < 0.01, ***p < 0.001 indicated significant differences, and "ns" indicated no significant difference. F: The heatmap demonstrated the differences in RiskScore, mRNA, estimate, cibersort, and xCell between High C2 Score and Low C2 Score. The color scale was based on standardized data. G: Bar plots presented the correlation analysis between immune cells and the constituent scoring genes and C2 Score. The color of the dots represented the magnitude of the p-value, and the size of the dots represented the correlation strength. *p ≤ 0.05, **p < 0.01, ***p < 0.001 indicated significant differences, and "ns" indicated no significant difference. H: The heatmap displayed the correlation analysis results between immune cells and the modeling genes, High C2 Score, Low C2 Score, and overall survival (OS).

To investigate variations in immune cell composition between the high and low C2 GJA4+ endothelial cell score groups, we employed the CIBERSORT algorithm (Fig. 4A). Box plots demonstrated that the low-score group exhibited increased proportions of CD8+ *T* cells, activated memory CD4+ *T* cells, M1 macrophages, plasma cells, and regulatory T cells (Tregs). In contrast, the high-score group showed elevated proportions of M2 and M0 macrophages (Fig. 4B-C).

The ESTIMATE package was utilized to compute StromalScore, ImmuneScore, ESTIMATEScore, and tumor purity for each group. The low-score group exhibited higher values for StromalScore, ImmuneScore, and ESTIMATEScore, while the high-score group showed higher tumor purity (Fig. 4D-E).

A heatmap was created to illustrate the variations in RiskScore, mRNA expression levels, ESTIMATE scores, CIBERSORT results, and xCell analysis between the two groups (Fig. 4F). A bar plot depicted the correlation between immune cells, model genes, and risk groups (Fig. 4G), while another heatmap demonstrated correlations between immune cells, risk, and OS (Fig. 4H).

### Enrichment analysis of differentially expressed genes between high and low C2 GJA4+ endothelial cell score groups

**Fig. 5:**
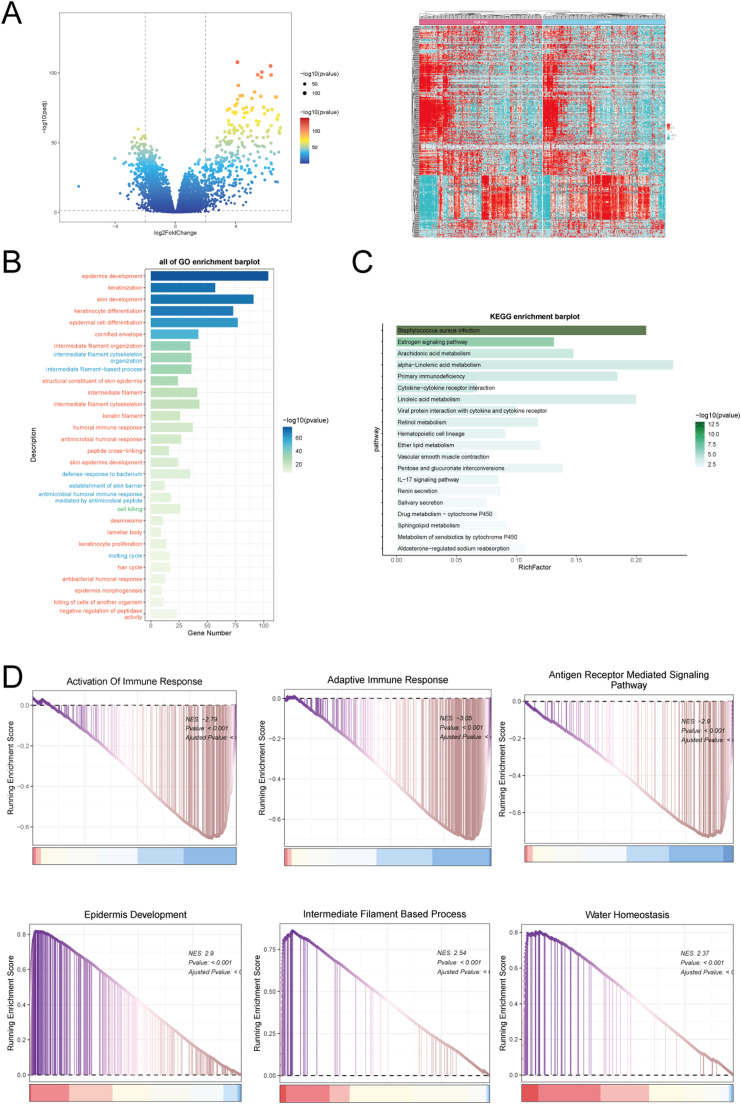
**Enrichment Analysis of Melanoma Endothelial Cells.** A: The volcano plot and heatmap depicted the expression profiles of differentially expressed genes between High C2 Score and Low C2 Score groups. B: The bar graph displayed the results of all enriched analyses for Gene Ontology (GO). C: The KEGG enrich *ent* analysis of differentially expressed genes demonstrated the enrichment results across different pathways. D: The GSEA enrichment analysis of Gene Ontology Biological Processes (GO-BP) terms for differentially expressed genes revealed the activity of different pathways in different groups.

The volcano plot and heatmap revealed significant differences in gene expression between the high and low C2 GJA4+ endothelial cell score groups (Fig. 5A). To clarify the functional roles of the differentially expressed genes, we performed enrichment analyses encompassing GO_BP, KEGG, and GSEA.

The GO_BP enrichment analysis identified significant associations with processes such as epidermis development, keratinization, skin development, keratinocyte differentiation, and epidermal cell differentiation in the low-score group (Fig. 5B). KEGG pathway analysis indicated notable enrichment in pathways related to Staphylococcus aureus infection and estrogen signaling in the low-score group (Fig. 5C). GSEA revealed significant enrichment of pathways related to immune response activation, adaptive immune response, and antigen receptor-mediated signaling in the low-score group. Conversely, the high-score group exhibited enrichment in pathways associated with epidermis development, intermediate filament-based processes, and water homeostasis (Fig. 5D).

### Genetic mutation analysis and drug sensitivity analysis

**Fig. 6:**
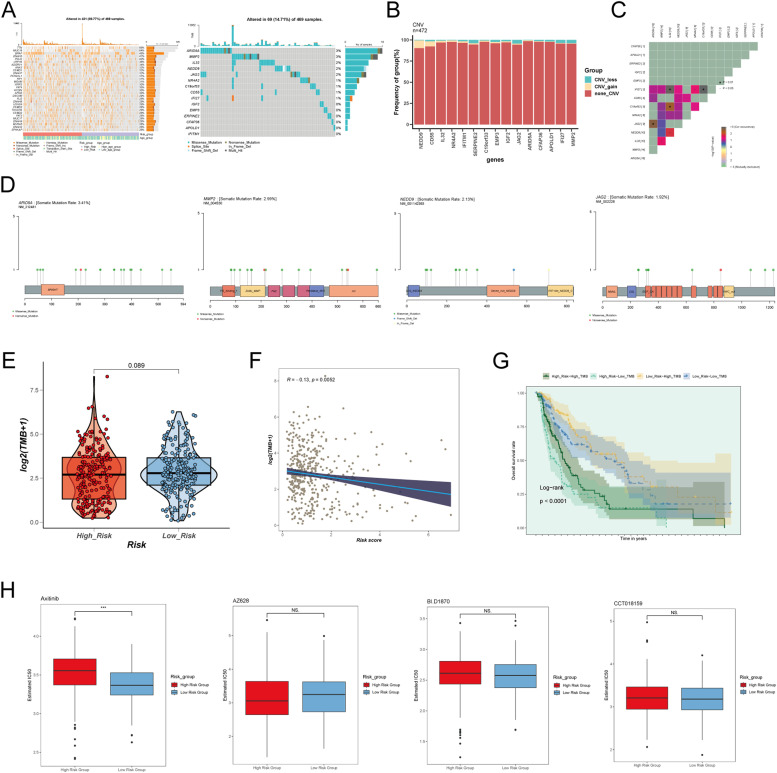
**Analysis of Gene Mutations and Drug Sensitivity in Melanoma Endothelial Cells** A: The waterfall plot of gene mutations displayed the mutation patterns of the top 30 genes in 421 individual cells and the mutations of the top 15 constituent modeling genes in 69 samples. The bar graph at the top represented the mutation burden of each sample, while the bar graph on the right represented the overall mutation frequency of the gene in these samples. B: Copy number variations of modeling genes were shown. Blue indicated chromosomal copy number loss, red indicated chromosomal copy number gain, and orange indicated no change in chromosomal copy number. C: The heatmap illustrated the correlation of mutations in the constituent modeling genes. D: The SNP plot visualized the mutation patterns of different genes. E: Box plots and violin plots displayed the differences in tumor mutational burden (TMB) between High C2 Score and Low C2 Score groups. The box plot showed the median (line within the box), upper and lower quartiles (box), and data range. *p ≤ 0.05, **p < 0.01, ***p < 0.001 indicated significant differences, and "ns" indicated no significant difference. F: The scatter plot depicted the correlation analysis between TMB and Risk Score. G: The survival curve presented the survival analysis results for High C2 Score-High TMB, High C2 Score-Low TMB, Low C2 Score-High TMB, and Low C2 Score-Low TMB groups. H: The box plot displayed the differences in drug sensitivity between High C2 Score and Low C2 Score groups. *p ≤ 0.05, **p < 0.01, ***p < 0.001 indicated significant differences, and "ns" indicated no significant difference.

We performed mutation analysis on melanoma samples, identifying TTN, MUC16, and BRAF as the most frequently mutated genes among the top 30 somatic mutations. Within the 15 modeling genes, ARID5A and MMP2 exhibited higher mutation frequencies (Fig. 6A). Copy number variation (CNV) analysis of these 15 genes showed that most had minimal CNV events, with NEDD9 and CD55 exhibiting relatively high frequencies of CNV_gain events. Conversely, SERPINE2, JAG2, IFI27, and MMP2 were associated with higher frequencies of CNV_loss events (Fig. 6B). A heatmap illustrating pairwise correlations among these genes confirmed frequent concurrent mutations (Fig. 6C), and SNP plots depicted mutation patterns for ARID5A, MMP2, NEDD9, and JAG2 (Fig. 6D).

The low C2 GJA4+ endothelial cell score group displayed a marginally higher tumor mutational burden (TMB) compared to the high-score group (Fig. 6E). Fig. 6F presents the correlation analysis between tumor mutational burden (TMB) and risk scores. Additionally, survival analysis across various risk and TMB categories—namely High C2 Score-High TMB, High C2 Score-Low TMB, Low C2 Score-High TMB, and Low C2 Score-Low TMB—was conducted, demonstrating significant differences in survival outcomes as depicted in Fig. 6G.

Finally, we evaluated chemotherapy drug sensitivity by comparing IC50 levels between high and low C2 GJA4+ endothelial cell score groups. Fig. 6H displays the IC50 values for four anticancer agents (Axitinib, AZ628, BI.D1870, CCT018159). Significant differences in IC50 levels were noted between the two groups. Notably, the high C2 GJA4+ endothelial cell score group showed lower IC50 values for AZ628, whereas the low C2 GJA4+ endothelial cell score group had lower IC50 values for Axitinib, BI.D1870, and CCT018159.

### Differential expression of GJA4

**Fig. 7:**
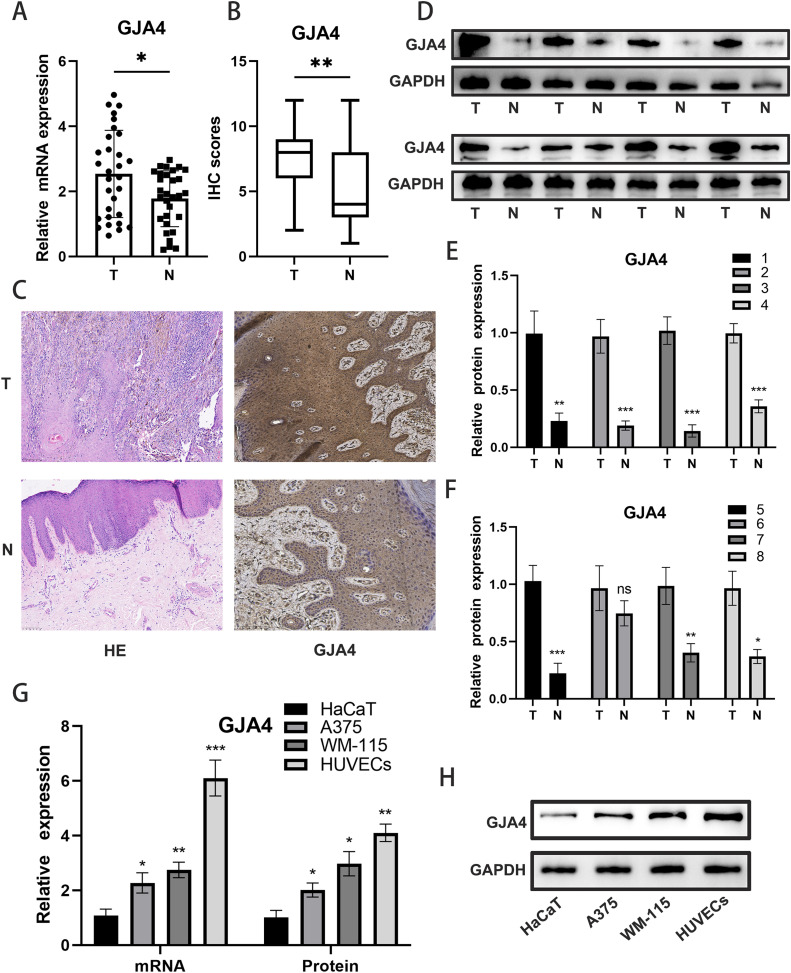
**Differential expression of GJA4 in cancerous and adjacent non-cancerous tissues.** A: Quantitative analysis of PCR (qPCR): GJA4 mRNA expression in 30 pairs of cancerous and adjacent non-cancerous samples, showing significant upregulation in tumor tissues. B, C:Immunohistochemistry (IHC) analysis of GJA4 expression in paired tissue samples, confirming elevated GJA4 protein levels in tumor tissues. D-F: Western blot analysis of GJA4 protein expression in eight pairs of tissue samples, corroborating the qPCR and IHC findings of increased GJA4 expression in tumor tissues. G-H: RNA and protein expression analysis of GJA4 in HaCaT, A375, WM-1115, and HUVECs cell lines, demonstrating slight upregulation in melanoma cell lines and highest expression in HUVECs cell lines.

To assess the expression of GJA4, we conducted qPCR analysis on 30 pairs of cancerous and adjacent non-cancerous tissue samples, revealing a significant upregulation of GJA4 mRNA in tumor tissues (Fig. 7A). Western blot analysis further confirmed the pronounced increase in GJA4 protein expression across eight pairs of tissue samples (Fig. 7D-F). These findings were corroborated by immunohistochemistry, which also demonstrated elevated GJA4 expression in tumor tissues (Fig. 7B-C). Bioinformatic analysis indicated that GJA4 expression is predominantly localized to endothelial cells. To validate this, we evaluated GJA4 expression at both RNA and protein levels in HaCaT, A375, WM-1115, and HUVECs cell lines. While a slight upregulation of GJA4 was observed in melanoma cell lines, the highest expression was detected in HUVECs (Fig. 7G-H).

### Functional validation of GJA4 knockout

**Fig. 8:**
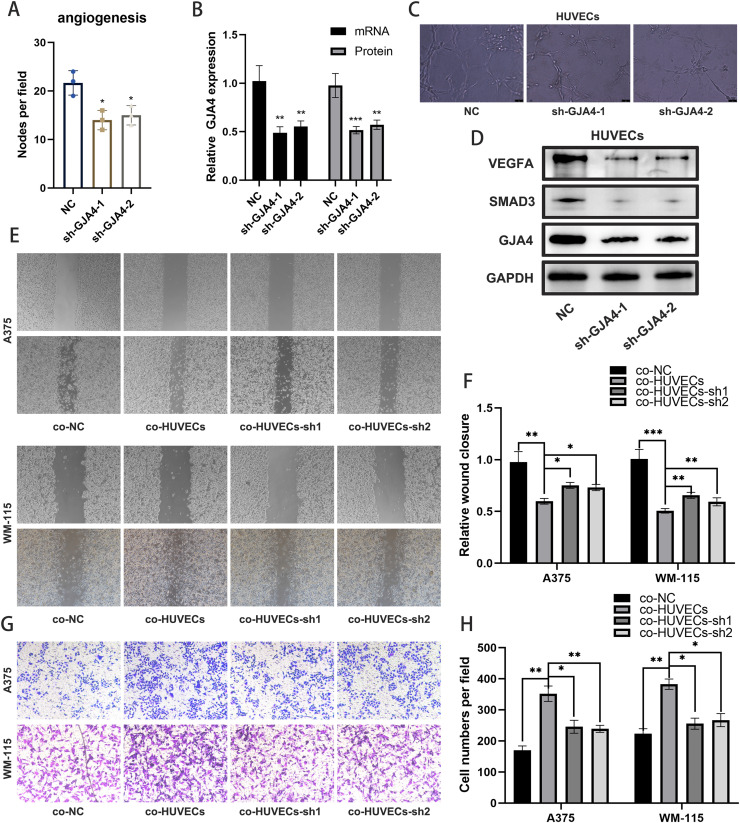
**Functional validation of GJA4 knockout in HUVECs.** A: Relative quantification of angiogenesis assays. B: Relative quantification of WB and q-RT PCR experiment. C: Angiogenesis assays showing that GJA4 knockout significantly inhibits the angiogenic capability of HUVECs. D: Measurement of VEGFA levels and TGF-β signaling pathway activity in HUVECs via WB experiment. E-H: Co-culture experiments with HUVECs and A375 or WM-115 melanoma cell lines, indicating that HUVECs enhance the invasion and metastasis of melanoma cells. This enhancement is significantly inhibited when melanoma cells are co-cultured with GJA4-knockout HUVECs.

We successfully knocked out GJA4 in HUVECs cell lines, with confirmation via qPCR and Western blot analysis (Fig. 8A-B). Angiogenesis assays revealed that GJA4 knockout significantly impaired the angiogenic capacity of HUVECs (Fig. 8C). This was accompanied by a reduction in VEGFA levels and suppression of the TGF-β signaling pathway (Fig. 8D). When co-culturing HUVECs with A375 and WM-115 melanoma cell lines, we observed that HUVECs enhanced the invasion and metastasis potential of the melanoma cells. However, co-culturing with GJA4-knockout HUVECs resulted in a marked inhibition of these abilities in melanoma cells (Fig. 8E-H). These results indicate that GJA4 derived from HUVECs significantly promotes the invasive and metastatic behavior of melanoma cells.

### In vivo functional validation of GJA4

**Fig. 9:**
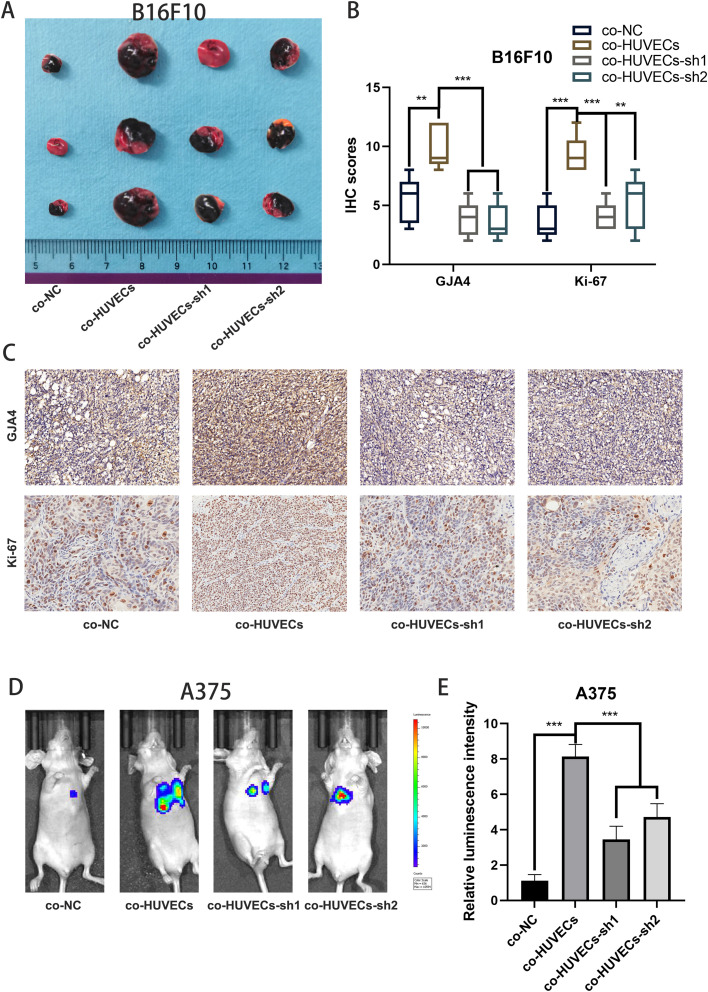
**In vivo functional validation of GJA4.** A: Subcutaneous tumor formation assay with B16F10 cells co-cultured with HUVECs. Tumor volume was significantly increased when B16F10 cells were co-cultured with HUVECs compared to GJA4-knockout HUVECs. B: Relative quantification of immunohistochemical test. C: Representative images of immunohistochemical test. D: Representative images of lung metastasis experiment. E: Relative quantification of lung metastasis experiment.

To substantiate our in vitro findings, we performed in vivo experiments. Subcutaneous tumor formation assays using B16F10 cells co-cultured with HUVECs demonstrated that co-culturing with HUVECs led to increased tumor volume and elevated Ki-67 expression. However, co-culturing with GJA4-knockout HUVECs significantly impaired the proliferation of B16F10 cells (Fig. 9A-C). Additionally, we assessed lung metastasis potential in A1375 cells co-cultured with HUVECs. The co-cultured A1375 cells exhibited enhanced lung metastasis, whereas co-culture with GJA4-knockout HUVECs significantly suppressed this capability (Fig. 9D-E). These results underscore the critical role of GJA4 in facilitating the proliferative and metastatic potential of melanoma cells through interaction with HUVECs.

### Exploration of GJA4 and the immune microenvironment

**Fig. 10:**
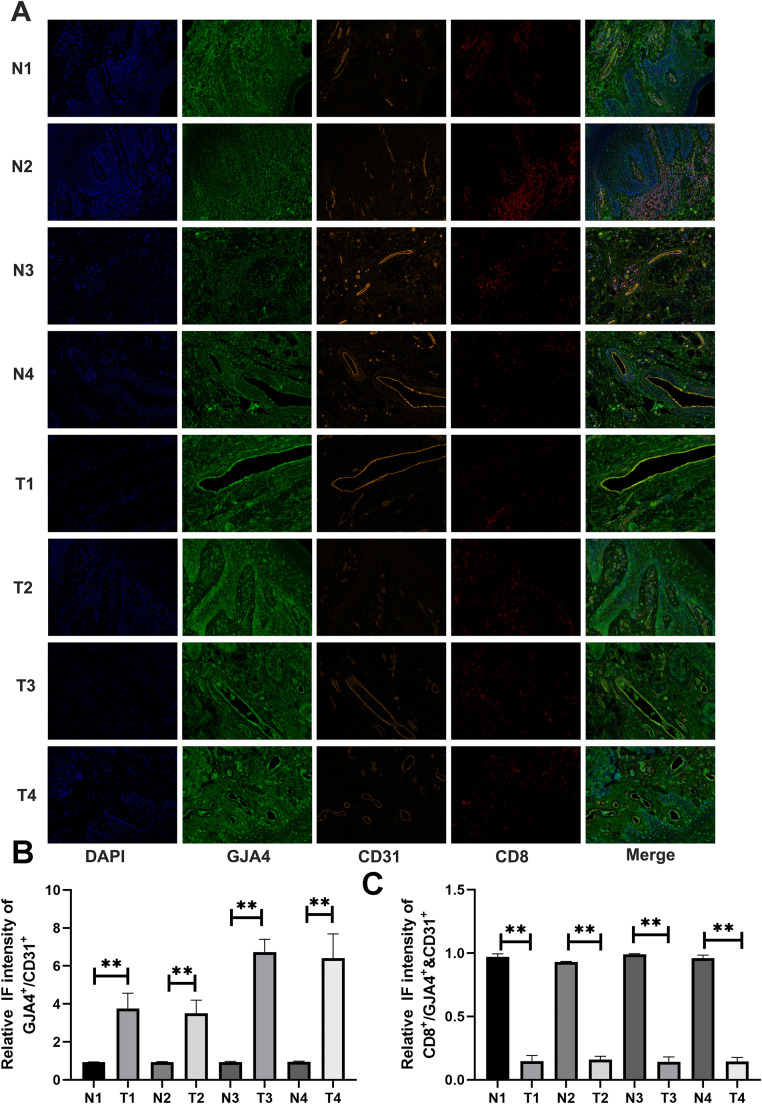
**Exploration of GJA4 and the immune microenvironment.** A: mIHC analysis shown that GJA4 expression was significantly higher in CD31-positive areas of tumor tissues compared to normal tissues. Regions with dual positivity for CD31 and GJA4 showed lower levels of CD8+ *T* cell infiltration. B-C: Relative quantification of mIHC.

To investigate the association between GJA4 expression and the immune microenvironment, we performed multiplex immunohistochemistry (mIHC) on four pairs of tumor tissues and their matched normal counterparts (Fig. 10A). Quantitative analysis of mIHC results revealed that GJA4 expression was significantly elevated in CD31-positive endothelial regions within tumor tissues compared to adjacent normal tissues (Fig. 10B). Moreover, areas with co-expression of CD31 and GJA4 exhibited markedly reduced infiltration of CD8⁺ T cells (Fig. 10C). These observations suggest that GJA4 expression in tumor-associated endothelial cells may contribute to the suppression of CD8⁺ T cell infiltration, thereby facilitating immune evasion within the tumor microenvironment.

## Discussion

Melanoma represents one of the most aggressive and lethal types of skin cancer, rendering its treatment a critical area of focus in medical research [1,25]. Although there has been progress in melanoma treatment over recent decades, the advancement of personalized medicine in this domain has remained relatively sluggish [26,27]. Personalized medicine aims to tailor treatment plans based on each patient's unique genomic profile, thereby improving therapeutic outcomes [28]. However, the high heterogeneity and complex genetic variation patterns of melanoma present significant challenges to achieving this goal [29,30]. In recent years, the rapid development of single-cell technologies has brought new hope to personalized medicine [31,32]. By comprehensively analyzing the genome, transcriptome, and epigenome of individual tumor cells, Researchers can achieve a deeper insight into tumor heterogeneity and evolution, thereby enabling the creation of more tailored and effective treatment strategies for individual patients. The application of these technologies is expected to accelerate the progress of personalized medicine in melanoma, enhance treatment efficacy, and improve patient prognosis.

Our study utilized single-cell RNA sequencing technology to uncover the developmental trajectories and intercellular communication networks of endothelial cell subtypes in melanoma, providing new perspectives and tools for advancing personalized medicine. Using the Slingshot method, we identified two differentiation trajectories of endothelial cell subtypes and detailed their distribution on UMAP plots. Notably, C2 GJA4+ endothelial cells demonstrated strong signaling capabilities, engaging in extensive signal exchange with other endothelial cell subtypes and forming close connections with tumor cells. This intercellular communication network helps elucidate the complexity and heterogeneity of melanoma. Through CellChat analysis, we further quantified the ligand-receptor interactions between endothelial cell subtypes and other cell types, highlighting the pivotal role of C2 GJA4+ endothelial cells in the melanoma microenvironment [33].

The research we performed not only identified marker genes closely associated with patient prognosis but also, through survival analysis and drug sensitivity analysis, determined the potential applications of these genes in personalized therapy. These findings offer new strategies for precision medicine in melanoma, potentially accelerating the development and implementation of personalized treatment plans, enhancing therapeutic efficacy, and improving patient survival outcomes [34,35].

The experiments we performed validated the critical role of GJA4 in melanoma, further supporting the findings from single-cell research and providing strong evidence for the advancement of personalized medicine. In 30 pairs of cancerous and adjacent non-cancerous tissues, qPCR analysis revealed a significant upregulation of GJA4 mRNA in tumor tissues, which was corroborated by Western blot and immunohistochemical analyses. These results align with existing literature, indicating the importance of GJA4 in the tumor microenvironment, particularly in promoting tumor cell proliferation and metastasis [36].

In cellular experiments, we further explored the function of GJA4 in melanoma cell lines. Knockout of GJA4 in HUVECs significantly inhibited their angiogenic capacity and reduced VEGFA levels and TGF-β signaling pathway activity [37]. These findings indicate that GJA4 may contribute to melanoma progression through the regulation of angiogenesis and critical signaling pathways. This aligns with prior research emphasizing the essential role of angiogenesis in tumor growth and metastasis [38,39].

In co-culture experiments with A375 and WM-115 melanoma cell lines, GJA4 knockout in HUVECs significantly inhibited the invasion and metastasis of melanoma cells. This finding further supports the role of GJA4 in tumor-endothelial cell interactions [40,41]. In vivo experiments further validated these findings. When B16F10 cells were co-cultured with HUVECs, tumor volume and Ki-67 expression increased, whereas co-culture with GJA4-knockout HUVECs significantly inhibited tumor proliferation. Additionally, lung metastasis experiments with A1375 cells showed that co-culture with GJA4-knockout HUVECs significantly suppressed their lung metastasis capability. These results demonstrate that GJA4 is vital for the interaction between tumor cells and endothelial cells, and its knockout can substantially reduce tumor growth and metastasis [42,43].

Multiplex immunofluorescence analysis also revealed that high GJA4 expression in tumor endothelial cells might influence the immune microenvironment by inhibiting CD8+ *T* cell infiltration. CD8+ *T* cells are crucial effector cells in the tumor immune response, with their levels of infiltration closely linked to patient outcomes. Our findings suggest that GJA4 may influence the tumor immune microenvironment by modulating CD8+ *T* cell infiltration. This highlights a potential new avenue for investigating how GJA4 contributes to tumor immune evasion [12].

In summary, our study not only elucidate the critical role of GJA4 in melanoma but also provide a new molecular target for the development of personalized therapeutic strategies. The role of GJA4 in promoting tumor cell proliferation, metastasis, and regulating the immune microenvironment indicates its significant potential in melanoma personalized medicine. These findings are expected to significantly advance the progress of personalized medicine in melanoma, improve therapeutic outcomes, and enhance patient prognosis. Future studies should investigate the regulatory mechanisms of GJA4 and its implications across various tumor types, potentially offering new perspectives for developing broad-spectrum cancer treatment strategies.

## Conclusions

Our study highlights the pivotal role of GJA4 in melanoma progression and its potential as a therapeutic target for personalized medicine. We demonstrated that GJA4 is significantly upregulated in melanoma tissues, promoting angiogenesis, proliferation, and metastasis. Knockout of GJA4 in HUVECs inhibited these processes, suggesting its crucial involvement in tumor growth and immune evasion. These findings highlight the potential of targeting GJA4 to advance personalized treatment approaches, increase therapeutic efficacy, and improve patient outcomes in melanoma. Future research should explore GJA4′s regulatory mechanisms and its role in other cancers to further validate its therapeutic potential.

## Funding

The present study was financially supported by the Scientific research project of universities in Anhui Province (Nos. 2022AH040163), the Open Project of Key Laboratory of Dermatology (Anhui Medical University), Ministry of Education (Nos.AYPYS2024-6), and Anhui Province key research and development program (Nos.2022e07020041).

## Data availability

Analyses were conducted using R (version 4.2.1) and GraphPad (GraphPad Prism 8.0). The codes used to support the findings of this study is available from the corresponding author on reasonable request.

## CRediT authorship contribution statement

**Yantao Ding:** Writing – review & editing, Writing – original draft, Visualization, Validation, Supervision, Software, Resources, Project administration, Methodology, Investigation, Funding acquisition, Formal analysis, Data curation, Conceptualization. **Si Xie:** Writing – review & editing, Writing – original draft, Visualization, Validation, Supervision, Resources, Project administration, Investigation, Formal analysis, Data curation, Conceptualization. **Wenyang Nie:** Writing – review & editing, Writing – original draft, Visualization, Validation, Software, Resources, Methodology, Investigation, Formal analysis, Data curation, Conceptualization. **Yun Bai:** Validation, Software, Project administration, Funding acquisition, Formal analysis. **Tianyu Yao:** Writing – review & editing, Writing – original draft, Visualization, Software, Resources, Investigation, Funding acquisition, Conceptualization. **Yixiao Wang:** Writing – review & editing, Writing – original draft, Supervision, Project administration, Investigation, Formal analysis, Conceptualization. **Jiajie Chen:** Writing – review & editing, Writing – original draft, Validation, Supervision, Project administration, Methodology, Funding acquisition, Data curation, Conceptualization. **Bo Liang:** Writing – original draft, Validation, Project administration, Conceptualization. **Yi Zhou:** Writing – original draft, Validation, Resources, Investigation, Data curation. **Hui Cheng:** Writing – review & editing, Writing – original draft, Validation, Project administration, Funding acquisition, Data curation. **Zaixing Wang:** Writing – review & editing, Writing – original draft, Visualization, Validation, Supervision, Resources, Investigation, Formal analysis, Data curation, Conceptualization. **Shengxiu Liu:** Writing – review & editing, Writing – original draft, Visualization, Validation, Supervision, Software, Resources, Investigation, Formal analysis, Data curation, Conceptualization.

## Declaration of competing interest

All authors declare no potential conflicts of interest.

## Appendix Supplementary materials

Image, application 1

Image, image 2

Image, application 3

Image, image 4

Image, image 5

Image, image 6

Image, image 7

Image, image 8

Image, application 9
