# Supplementary material for: Integrated single-cell sequencing for the development of a GJA4-based precision immuno-prognostic model in melanoma
Source: Transl Oncol. 2025 Jul 9;59:102450. doi: 10.1016/j.tranon.2025.102450 (PMC12275486; doi:10.1016/j.tranon.2025.102450)
Supplement: Supplementary file 1 [file mmc1.docx]

**Authorship Change Statement**

We would like to request a change in the authorship order of our manuscript entitled " **Integrated Single-Cell Sequencing for the Development of a GJA4-Based Precision Immuno-Prognostic Model in Melanoma**" (Manuscript ID: **TRANON-D-24-03476**) due to the substantial contributions made during the revision process.

Specifically:

- **Sixie** has been actively involved in the extensive revision of the manuscript, particularly in correcting data inconsistencies and rewriting key sections of the manuscript. In recognition of her significant contributions during the resubmission phase, we propose to move her name forward by one position, exchanging places with **Huanghe**.
- **Shengxiu Liu** also contributed significantly to the revision process, including extensive data correction and manuscript refinement. After mutual discussion and agreement among all co-authors, we propose to move her name one position backward, exchanging places with **Zaixing Wang**.

Both Sixie and Shengxiu Liu played important roles in ensuring the scientific accuracy and clarity of the revised manuscript through their dedicated efforts in data verification and content rewriting.

All authors have been informed of and agreed to this change in authorship order.
