# Supplementary figures and images for: Integrated single-cell sequencing for the development of a GJA4-based precision immuno-prognostic model in melanoma
Source: Transl Oncol. 2025 Jul 9;59:102450. doi: 10.1016/j.tranon.2025.102450 (PMC12275486; doi:10.1016/j.tranon.2025.102450)

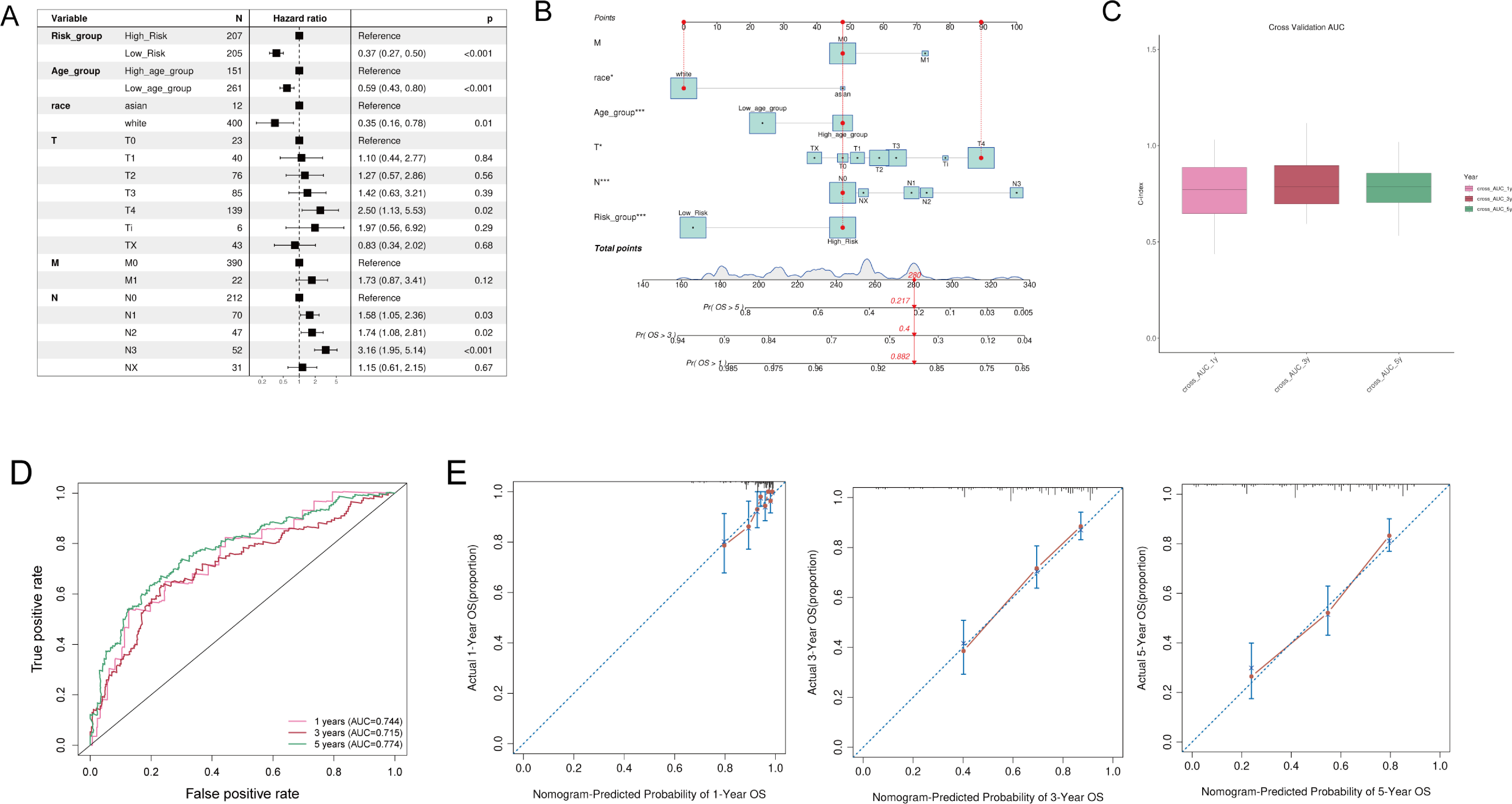

Supplement: Supplementary file 2 [file mmc2.jpg]

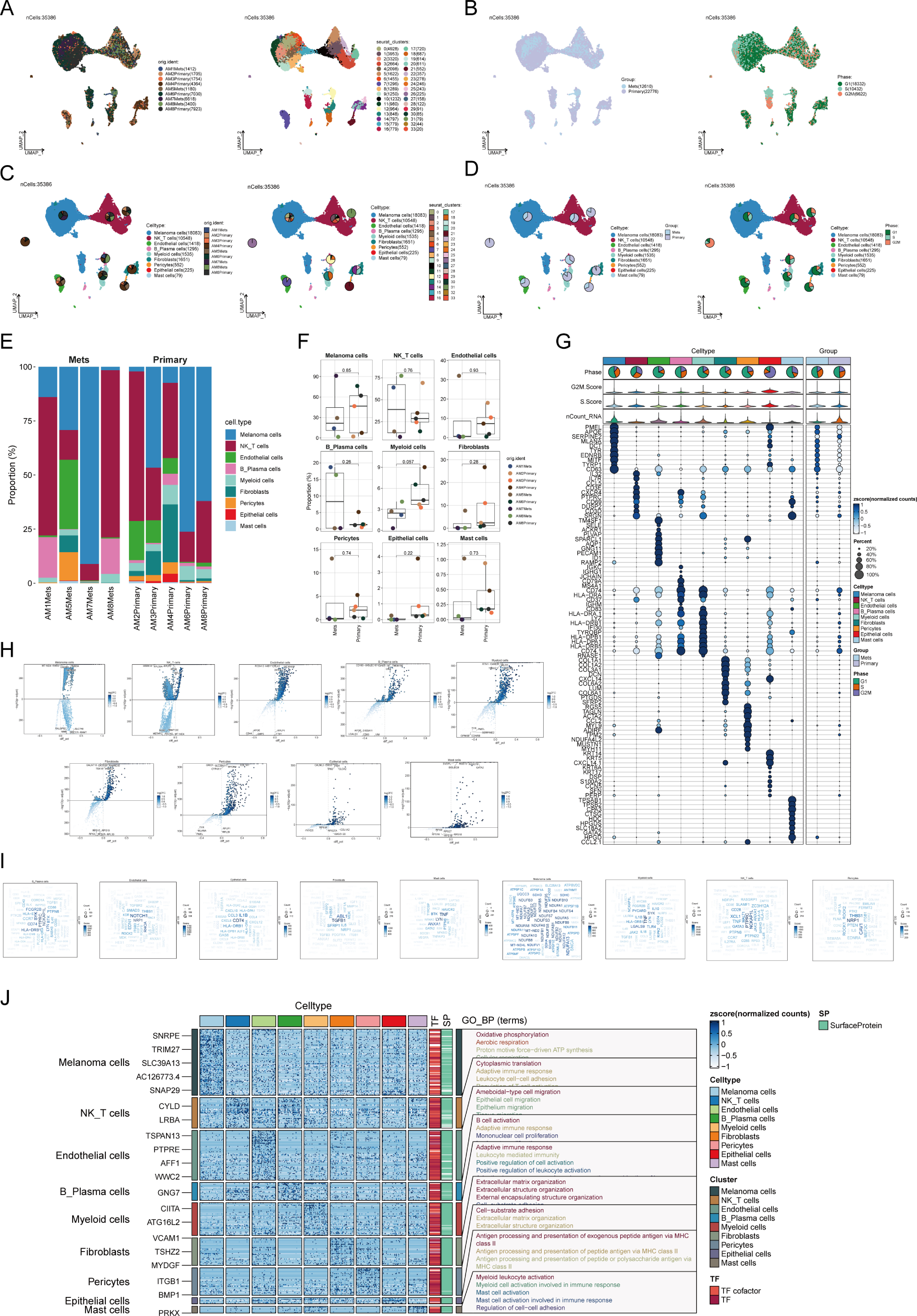

Supplement: Supplementary file 4 [file mmc4.jpg]

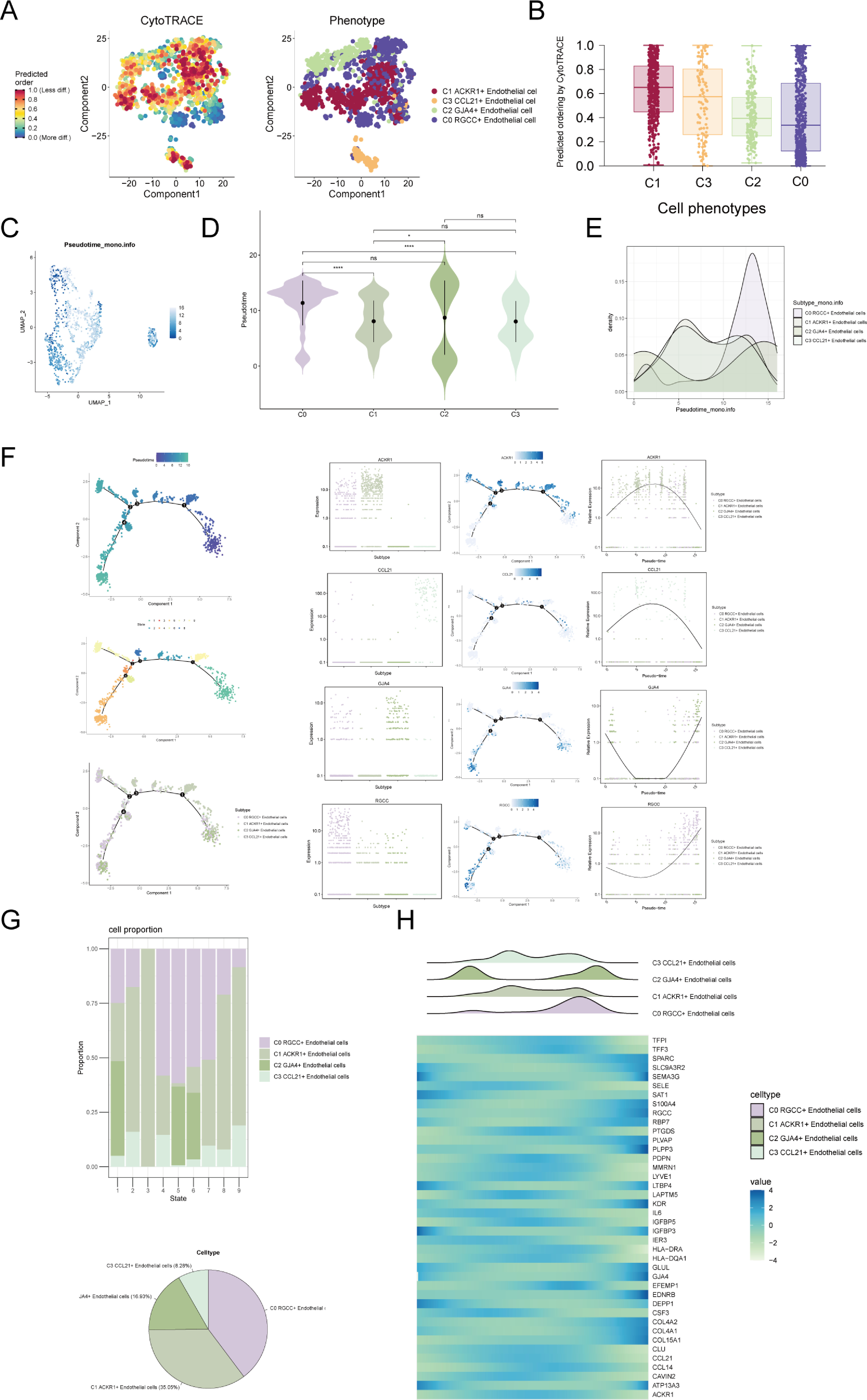

Supplement: Supplementary file 5 [file mmc5.jpg]

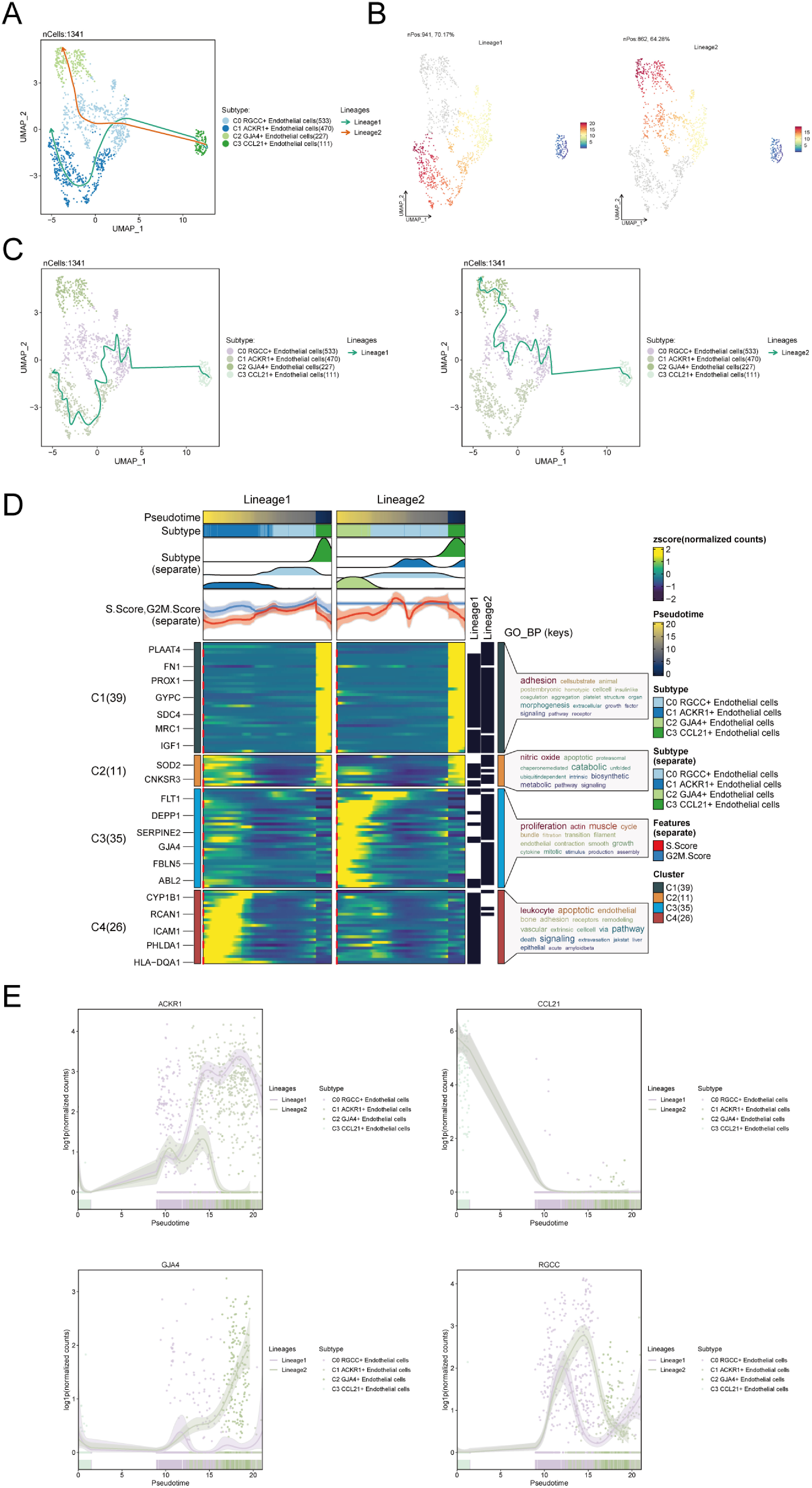

Supplement: Supplementary file 6 [file mmc6.jpg]

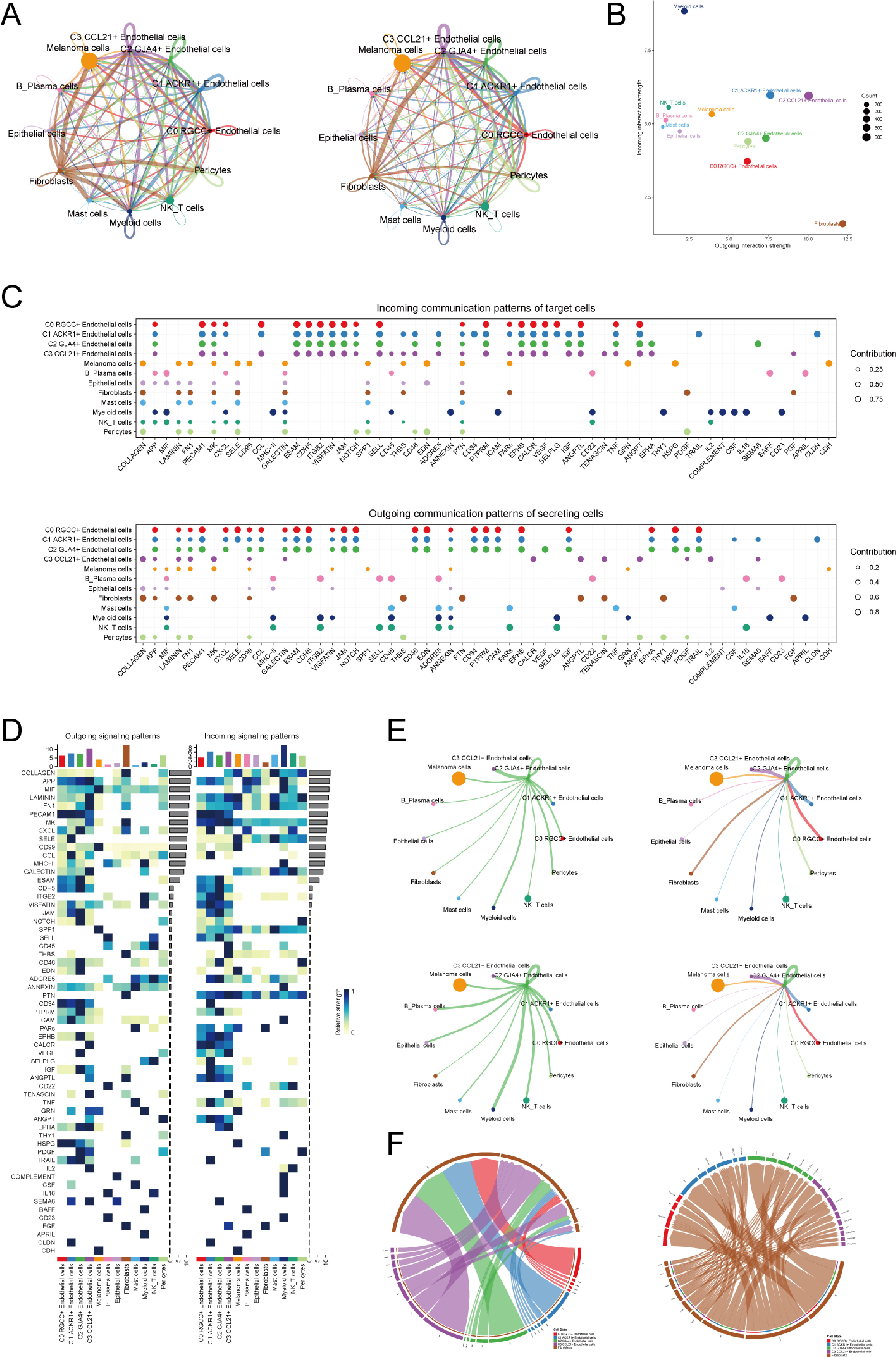

Supplement: Supplementary file 7 [file mmc7.jpg]

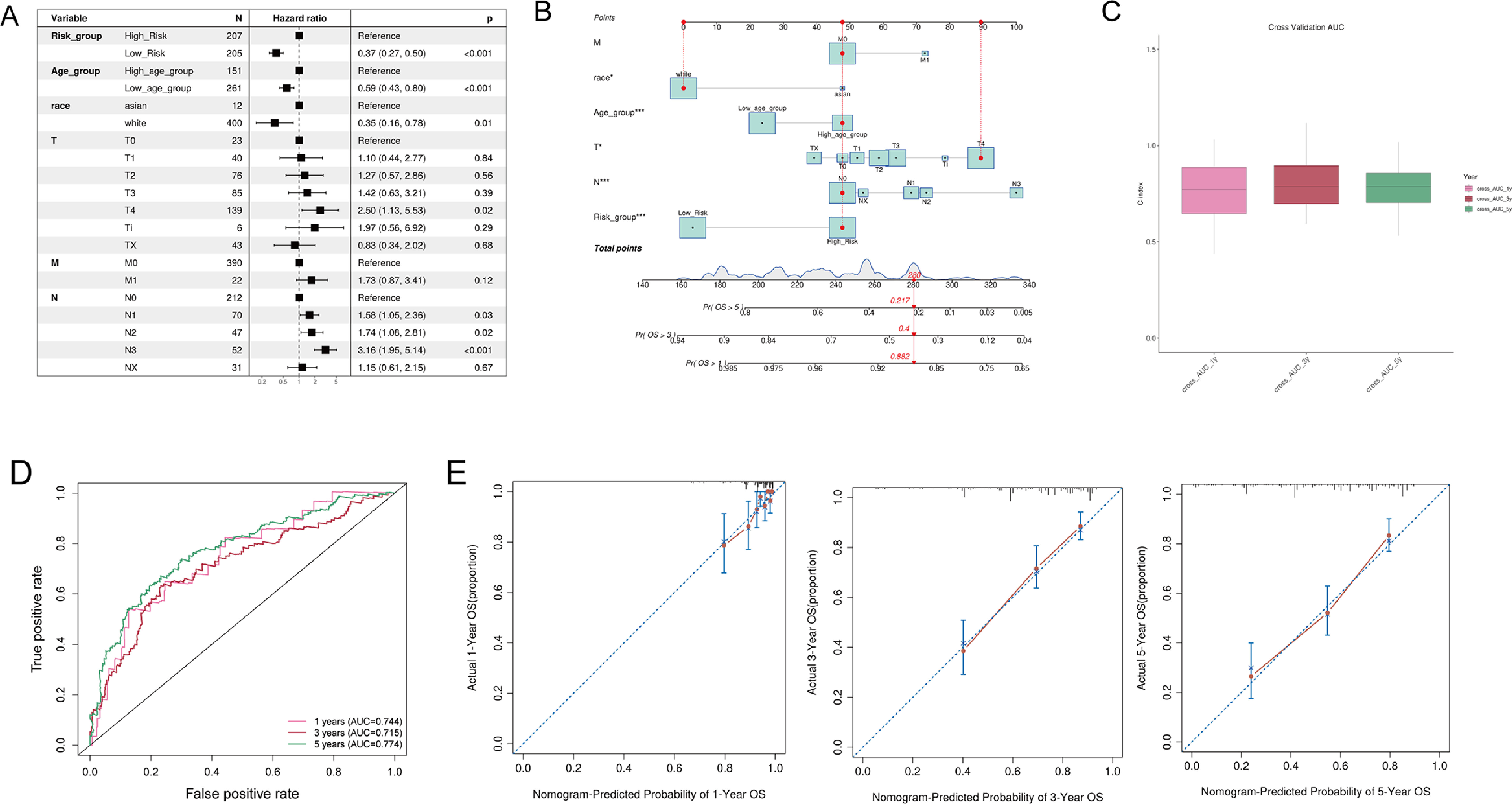

Supplement: Supplementary file 8 [file mmc8.jpg]
