# Supplementary material for: Integrated single-cell sequencing for the development of a GJA4-based precision immuno-prognostic model in melanoma
Source: Transl Oncol. 2025 Jul 9;59:102450. doi: 10.1016/j.tranon.2025.102450 (PMC12275486; doi:10.1016/j.tranon.2025.102450)
Supplement: Supplementary file 3 [file mmc3.docx]

**Supplementary Figure 1: Cell Type Identification in Melanoma**

A: UMAP visualization showcased the distribution of 35,386 cells based on their tissue origins (left) and cell clusters (right).

B: UMAP visualization exhibited the distribution of cell tissue types (left) and cell cycle stages (right).

C: UMAP visualization displayed the distribution of sample origins for nine cell types (left) and classification by the Seurat method (right).

D: UMAP visualization illustrated the distribution of different cell tissue types (left) and cell cycle stages (right) for nine cell types.

E: Bar chart displayed the proportion of nine cell types across different tissue origins.

F: Box plot highlighted the proportion of tissue origins for different cell types among the nine identified ones.

G: Bubble plot showed the differential expression of the top 10 marker genes for the nine cell types. The color of the bubbles represented normalized counts, while the size represented the proportion of gene expression.

H: Volcano plot presented the expression pattern of differentially expressed genes among the nine cell types.

I: Word cloud displayed the frequency of gene enrichment for different genes among the nine cell types.

J: Heatmap showed the top 5 enriched Gene Ontology Biological Process (GO-BP) terms for differentially expressed genes among the nine cell types.

**Supplementary Figure 2: CytoTrace and Pseudo-Time Trajectory Analysis of Endothelial Cell Subpopulations**

A: UMAP visualization showcased the CytoTrace results of endothelial cell subpopulations. The color represented the varying degrees of differentiation (left).

B: Box plot displayed the predicted ordering by CytoTRACE. It is evident from the graph that C1 ACKR+ endothelial cells exhibited the lowest degree of differentiation, while C0 RGCC+ endothelial cells demonstrated the highest degree of differentiation.

C-E: UMAP visualization, violin plot, and ridge plot demonstrated the pseudo-time trajectory results of endothelial cell subpopulations. *p≤0.05, **p<0.01, ***p<0.001 indicated significant differences, while ns indicated no statistical difference.

F: Two-dimensional plot (left) showcased the derivation process of endothelial cells, while the trajectory of marker genes for endothelial cell subpopulations is depicted on the right. The curves represent fitted gene expression profiles, and the scatterplot colors represent subpopulation types. The two-dimensional plot in the middle exhibited the pseudo-time trajectory of different subpopulation marker genes.

G: Bar chart displayed the proportion of the four endothelial cell subpopulations in different pseudo-time stages (state1-state9) (top). The pie chart displayed the overall proportion of the four endothelial cell subpopulations among all endothelial cells (bottom).

H: Heatmap showed the expression levels of the top 10 marker genes for each of the four endothelial cell subpopulations along the trajectory.

**Supplementary Figure 3: Slingshot Analysis of Endothelial Cell Subpopulations**

A-C: UMAP visualizations presented two differentiation trajectories fitted by pseudo-time order for endothelial cell subpopulations (A, C). Another UMAP visualization (B) demonstrated the distribution of these two differentiation trajectories within the endothelial cells. Solid lines indicated the differentiation trajectories, with arrows indicating the direction of differentiation (from immature to mature). Lineage1: C3→C0→C1, Lineage2: C3→C0→C2.

D: Heatmap displayed the Gene Ontology Biological Process (GO_BP) enrichment analysis results for the two pseudo-time trajectories of endothelial cell subpopulations.

E: Scatter plots exhibited the expression changes of the marker genes for the four endothelial cell subpopulations along the two differentiation trajectories (Lineage1, Lineage2) in pseudo-time order. The curves represented gene expression profiles fitted based on standardized data, while the scatterplot colors represented different endothelial cell subpopulations.

**Supplementary Figure 4: Analysis of Communication between Endothelial Cells and All Cells**

A: The strength circle plot (left) and quantity circle plot (right) showcased the interactions between endothelial cell subgroups and all cells.

B: The scatter plot displayed the network analysis of all cell pathways, with different cell types represented by colors, and the size of dots representing cell quantities.

C: The bubble plots presented the patterns of cell communication between all cells, showing the incoming contribution (left) and outgoing contribution (right). The color of dots represented different cell types, while the size of dots indicated the cell quantity.

D: The heatmap showed the cell-cell interactions in terms of outgoing interactions (left) and incoming interactions (right). The different colors on the x-axis represented different cell types, and the varying shades of colors indicated the strength of the signals.

E: The quantity circle plots (left) and strength circle plots (right) depicted the interactions between C2 GJA4+ endothelial cells as a source and all other cells, as well as the interactions between C2 GJA4+ endothelial cells as a target and all other cells.

F: The chord diagrams visualized the interactions between endothelial cells as a source and fibroblast cells (left), and the interactions between endothelial cells as a target and fibroblast cells (right).

**Supplementary Figure 5** **The model analysis.**

A: The forest plot presented the hazard ratios for High C2 Score, Low C2 Score, different age groups, different races, and different TNM stages.

B: The nomogram model predicted the differences in age groups, races, High C2 Score, Low C2 Score, and TNM stages.

C: The box plot displayed the visualized indices of 1, 3, and 5-year cross-validation. The box plot conveyed the median (line within the box), upper and lower quartiles (box), and data range.

D: The ROC curve depicted the AUC values for 1-year, 3-year, and 5-year predictions. (1-year AUC = 0.744, 3-year AUC = 0.715, 5-year AUC = 0.774). AUC1-year survival rate: 0.744, AUC3-year survival rate: 0.715, AUC5-year survival rate: 0.774.

E: The line plots presented the calibration curves for 1-year, 3-year, and 5-year predictions compared to the actual survival time using the Nomogram model.
