# Supplementary material for: Integrated single-cell sequencing for the development of a GJA4-based precision immuno-prognostic model in melanoma
Source: Transl Oncol. 2025 Jul 9;59:102450. doi: 10.1016/j.tranon.2025.102450 (PMC12275486; doi:10.1016/j.tranon.2025.102450)
Supplement: Supplementary file 9 [file mmc9.docx]

| **Oligonucleotides** | **Nucleotide sequence (5'-3')** |
| --- | --- |
| **siRNA** |  |
| Si-GJA4-1 | ACCCTCTTTCCATGGAATCCTTGTA |
| Si-GJA4-2 | CCCTCTTTCCATGGAATCCTTGTAA |
| **ShRNA**  Sh-GJA4-1  Sh-GJA4-2 | AGGCGGGAGGAATACTATTGT  GGCGGGAGGAATACTATTGTT |
| **Primer** |  |
| GAPDH | GGCCTCCAAGGAGTAAGACC (forward) |
|  | AGGGGAGATTCAGTGTGGTG (reverse) |
| GJA4 | CAGACCTGGCTGAGAGTGGA (forward) |
|  | AGGCGGTGAAAGTGGTGATA (reverse) |
|  |  |

**Table S1. Oligonucleotides used in research**
